# Effectiveness of Digital Health Interventions for Improving Antiretroviral Therapy Outcomes in People With HIV: Meta-Analysis and Trial Sequential Analysis of Randomized Controlled Trials

**DOI:** 10.2196/81019

**Published:** 2026-07-23

**Authors:** Pan Liu, Jiahao Meng, Xi Li, Yilin Xiong, Xuanyu Wang, Yuqing Xiang, Shuguang Gao

**Affiliations:** 1 Department of Orthopaedics, Xiangya Hospital, Central South University, No. 87 Xiangya Road, Changsha, Hunan, 410008, China, 86 13875980341

**Keywords:** digital health interventions, HIV, viral suppression, adherence, meta-analysis, mobile health, trial sequential analysis

## Abstract

**Background:**

Digital health interventions (DHIs) are increasingly used to support antiretroviral therapy (ART) management among people living with HIV. However, existing systematic reviews have largely focused on single intervention types or limited outcomes, and few have integrated multiple DHI modalities across both behavioral and clinical end points. Additionally, previous evidence has rarely incorporated analytical approaches, such as prediction intervals (PIs) or trial sequential analysis (TSA), leaving uncertainty regarding the robustness and generalizability of findings.

**Objective:**

This systematic review aimed to evaluate the effectiveness of DHIs in improving ART-related outcomes among people living with HIV.

**Methods:**

We systematically searched PubMed, Cochrane Library, Embase, and Web of Science for randomized controlled trials (RCTs) published up to February 29, 2026. Eligible studies included people living with HIV receiving ART and evaluated DHIs, such as SMS, mobile apps, phone calls, adherence monitoring devices, multimedia education, or multicomponent interventions. Outcomes included viral suppression, CD4^+^ cell count, adherence, and retention. Random-effects meta-analyses were conducted using restricted maximum likelihood estimation with Hartung-Knapp-Sidik-Jonkman adjustment. Effect sizes were reported as risk ratios (RRs) or mean differences (MDs) with 95% CIs and 95% PIs. TSA was performed to assess the sufficiency of cumulative evidence. A frequentist network meta-analysis was conducted to compare the relative effectiveness of different DHIs.

**Results:**

A total of 64 RCTs involving 22,286 participants were included. Compared with standard of care (SOC), DHIs improved subjective adherence (RR 1.13, 95% CI 1.04‐1.23; 95% PI 0.82‐1.57) and retention (RR 1.06, 95% CI 1.01‐1.12; 95% PI 0.81‐1.39). Viral suppression was modestly improved (RR 1.04, 95% CI 1.01‐1.07; 95% PI 0.96‐1.13), while no significant effect was observed for CD4^+^ cell count and objective adherence. TSA indicated sufficient evidence for viral suppression and subjective adherence but inconclusive evidence for other outcomes. In the network meta-analysis, SMS, mobile apps, and multicomponent interventions demonstrated statistically significant benefits versus SOC; however, all PIs crossed the null. Although phone calls ranked highest by surface under the cumulative ranking curve (SUCRA), differences between interventions were not robust.

**Conclusions:**

In contrast to previous systematic reviews that focused on single intervention types or limited outcomes, this systematic review provides a comprehensive synthesis of multiple outcomes across all types of DHIs, supporting their potential role as nonpharmacological strategies in HIV care. However, the wide 95% PI, together with a high risk of bias, small-study effects, and low to very low certainty of evidence based on GRADE (Grading of Recommendations Assessment, Development, and Evaluation), indicate substantial uncertainty regarding the true effects in future settings. Therefore, these findings should be interpreted with caution. These findings have important practical implications, as they may directly help inform the design of more targeted and context-specific digital interventions and highlight the need for further research to identify optimal implementation strategies in routine HIV care.

## Introduction

HIV infection remains a major global public health challenge [1]. The widespread use of antiretroviral therapy (ART) has substantially improved survival among people living with HIV and has gradually transformed HIV infection into a chronic condition that can be managed over the long term [2,3]. Successful ART is typically reflected in sustained viral suppression, immune recovery, reduced HIV-related morbidity and mortality, and a lower risk of transmission [4,5]. Although ART outcomes are influenced by multiple factors, including early diagnosis, timely treatment initiation, and health system support, patient-level adherence and retention remain central to treatment success [5]. Poor adherence is closely associated with virologic failure, drug resistance, disease progression, and increased mortality risk [6,7]. Therefore, the long-term support of ART management continues to be a core issue in HIV care.

In response to these ongoing challenges, digital health interventions (DHIs) have been increasingly used to support HIV care. DHIs use information and communication technologies to support disease management and the functioning of health systems [8]. These interventions take multiple forms, including patient-focused tools such as SMS text reminders, mobile apps, and electronic medication monitoring devices, as well as system-level technologies such as telemedicine platforms, electronic health records, and clinical decision support systems [8,9]. With the widespread global adoption of smartphones and internet connectivity, digital platforms provide an important foundation for expanding support for HIV care [10].

Compared with facility-based care alone, digital interventions may support ART management by enabling reminders, remote follow-up, and ongoing patient-provider communication [11,12]. In addition, digital approaches can help mitigate structural barriers to HIV care, such as geographic distance, transportation difficulties, stigma, and limited health care resources [13,14]. Consequently, digital technologies may not only improve access to care in resource-limited settings but also help reduce disparities in access to HIV services among rural and underserved populations in middle- and high-income countries [15,16].

Despite these potential advantages, an increasing number of randomized controlled trials (RCTs) have evaluated DHIs in HIV care, but the findings remain inconsistent. Available evidence suggests that various DHIs, including SMS text reminders, mobile apps, and multicomponent digital interventions, may improve ART-related outcomes [17,18]. These interventions vary widely in format, ranging from simple communication tools, such as SMS text, to more complex interactive platforms designed to support patient management and behavior change. Some studies have reported that tailored SMS text interventions and interactive platforms can improve medication adherence and virological outcomes in specific populations, such as adolescents and young adults living with HIV. However, other studies have found limited or no significant effects, indicating that the effectiveness of DHIs may depend on factors such as intervention design, implementation context, and population characteristics [19].

Existing systematic reviews and meta-analyses have attempted to synthesize the growing body of evidence on DHIs in HIV care. However, several important limitations remain. First, many previous reviews have focused on single types of interventions, most commonly SMS-based approaches, which limits the ability to compare the relative effectiveness of different digital modalities [20,21]. Second, previous studies have often examined a narrow set of outcomes, typically focusing on adherence, without simultaneously evaluating clinical outcomes such as viral suppression and immunological recovery [20-22]. Third, most meta-analyses have relied primarily on pooled effect estimates and CIs, with limited consideration of between-study variability and real-world applicability [20-24].

Furthermore, few studies have integrated multiple analytical frameworks to comprehensively evaluate both the magnitude and the robustness of intervention effects. Network meta-analysis provides an opportunity to compare multiple intervention types simultaneously by incorporating both direct and indirect evidence, which is particularly valuable in contexts where head-to-head comparisons between interventions are limited [25,26]. Although network meta-analysis has been increasingly adopted in comparative effectiveness research, its application in evaluating DHIs for HIV-related outcomes remains relatively limited.

Given these methodological gaps, a comprehensive and methodologically rigorous synthesis is warranted to assess not only the average effects of DHIs, but also the uncertainty, heterogeneity, and comparative effectiveness of different intervention strategies. Approaches such as prediction intervals (PIs) and network meta-analysis may offer a more informative assessment of how effects vary across settings and how different digital interventions compare with one another [27,28]. In turn, such evidence may better support clinical interpretation and inform the development and implementation of future digital HIV care programs.

Therefore, this systematic review aimed to provide an updated and comprehensive evaluation of the effectiveness of DHIs in improving ART-related outcomes among people living with HIV. We included the most recent RCTs and assessed multiple outcomes, including viral suppression, CD4^+^ cell counts, adherence, and retention in care. To enhance the robustness and interpretability of the findings, we applied a combination of analytical approaches, including pairwise meta-analysis, network meta-analysis, PI, and trial sequential analysis (TSA). By integrating these methods, this systematic review seeks to provide a more complete understanding of both the potential benefits and the limitations of DHIs, as well as their applicability across different real-world settings.

## Methods

### Protocol and Registration

This systematic review was reported in accordance with the PRISMA (Preferred Reporting Items for Systematic Reviews and Meta-Analyses) 2020 statement and PRISMA-S (Preferred Reporting Items for Systematic Reviews and Meta-Analyses literature search extension) to strengthen transparency [29,30]. The review protocol was registered on the PROSPERO International Prospective Register of Systematic Reviews (CRD42024567903). No major deviations from the registered study protocol occurred during the conduct of the review.

### Eligibility Criteria

Study selection was conducted according to the population, intervention, comparator, outcomes, and study design framework. The inclusion criteria were as follows: (1) population: people living with HIV receiving ART; (2) intervention: DHIs (eg, SMS text reminders, adherence monitoring devices, mobile apps, or multimedia education); (3) comparator: standard of care (SOC) or other DHIs; (4) outcomes: treatment-related outcomes, adherence, or retention; and (5) study design: RCTs. The exclusion criteria were as follows: non-RCTs and studies for which the full text was not available. No additional restrictions were applied.

### Information Sources and Search Strategy

We conducted a systematic search of 4 electronic databases—PubMed, Embase, Cochrane Library, and Web of Science. In addition, 2 reviewers (PL and JM) independently screened the reference lists of published systematic reviews and meta-analyses on DHIs in HIV care to identify eligible RCTs that might have been missed in the electronic database search.

The search strategy combined free-text terms and MeSH related to HIV, DHIs, and their synonyms. A specific search strategy was developed for this systematic review and adapted for each database based on previously published studies in this field. The complete search strategy is in the appendix. No restrictions were applied regarding publication date or language, and no additional methodological filters or search limits were used. The initial search was conducted on May 1, 2024, and the final search was updated to February 29, 2026. No attempts were made to obtain additional information by contacting study authors, experts, or other stakeholders.

### Selection Process

All retrieved records were imported into the Rayyan online platform for deduplication and screening. Moreover, 2 reviewers (PL and JM) independently screened the titles and abstracts of all records and conducted full-text assessments of studies considered potentially eligible. Where there were disagreements between reviewers about the inclusion of a paper, a consensus was reached through discussion among all authors.

### Data Collection Process and Data Items

A standardized data extraction form was developed in accordance with the Cochrane Handbook for Systematic Reviews of Interventions, and a pilot extraction was performed on a subset of included studies before formal data extraction. The following information was extracted: author, year of publication, trial registration number, study region, study population, study design, sample size, sex distribution, age (mean and SD), follow-up duration, outcome measures, and characteristics of the interventions. Data extraction was conducted independently by 2 reviewers (PL and JM), and any discrepancies were resolved through discussion with a third reviewer (SG). The primary outcomes of this systematic review were viral suppression and the final CD4^+^ cell count. Secondary outcomes included medication adherence and retention. Viral suppression was defined as an HIV viral load of <400 copies/mL. In this systematic review, adherence was defined as “good adherence,” corresponding to a medication adherence rate >90%. Adherence was classified as subjective or objective. Subjective measures were based on patient self-report, whereas objective measures were assessed by clinicians or trained personnel or obtained using electronic adherence monitoring devices. Retention was defined as the proportion of participants who remained engaged in care and were not lost to follow-up during the study period.

For multiarm studies in which multiple intervention groups shared the same control group and were included in the same meta-analysis, we followed the Cochrane Handbook recommendation to avoid double-counting by splitting the shared control group across the relevant comparisons [31]. For dichotomous outcomes, the number of events and total sample size in the shared control group were divided equally across comparisons; for continuous outcomes, the mean and SD were retained, while the sample size was divided equally. Decimal values were retained for effect-size calculation when necessary.

DHIs were defined according to the World Health Organization classification of digital health technologies [8]. Based on their mode of delivery and key characteristics, the included DHIs were classified into six mutually exclusive categories: (1) SMS, defined as short message service reminders or bidirectional messaging; (2) adherence monitoring devices, such as smart pill bottles or electronic dose monitoring systems; (3) mobile apps designed to support ART adherence or HIV care; (4) phone calls, including voice calls or telecounseling delivered via telephone; (5) multimedia education, defined as educational interventions delivered through web-based platforms or mobile devices using multimedia formats such as videos, animations, audio, or interactive learning modules; and (6) multiple digital interventions, which combined 2 or more digital modalities (eg, SMS text plus monitoring devices or mobile apps).

According to the process evaluation framework of the Medical Research Council, reach was defined as the proportion of the intended target population that was reached by or exposed to the intervention [32]. Uptake was defined as the reported adoption or use of the intervention or health promotion program [33]. Feasibility was defined as the practicality of implementing the intervention or program, typically assessed through indicators such as acceptability, adherence, potential cost-effectiveness, or the capacity of providers to deliver the intervention [34,35].

### Risk-of-Bias and Certainty Assessment

The risk of bias in the included RCTs was assessed using the Risk of Bias 2 (RoB 2) tool. RoB 2 evaluates potential sources of bias across five key domains: (1) the randomization process, (2) deviations from intended interventions, (3) missing outcome data, (4) measurement of the outcome, and (5) selection of the reported result [36]. Each domain was judged as presenting a “low risk of bias,” “some concerns,” or “high risk of bias,” and an overall risk-of-bias judgment was assigned for each study. The certainty of evidence for each outcome was assessed using the GRADE (Grading of Recommendations Assessment, Development, and Evaluation) approach with the online GRADEpro Guideline Development Tool (GRADEpro GDT) [37]. The assessment domains included risk of bias, inconsistency, indirectness, imprecision, and publication bias. The certainty of evidence was rated as high, moderate, low, or very low. Moreover, 2 reviewers (PL and JM) independently conducted the assessments, and any disagreements were resolved through consensus.

### Data Analysis

Pairwise meta-analyses were first conducted to evaluate the effectiveness of DHIs compared with control conditions. For dichotomous outcomes, risk ratios (RRs) with 95% CIs were calculated. For continuous outcomes, pooled estimates were expressed as mean differences (MDs) with 95% CIs. Given the anticipated clinical and methodological heterogeneity across studies, all analyses were performed using random-effects models, with between-study variance estimated by the restricted maximum likelihood method. CIs were adjusted using the Hartung-Knapp-Sidik-Jonkman approach [38]. When at least 10 studies were available, 95% PIs were calculated to estimate the range of effects expected in future studies [39]. Subgroup analyses were performed according to intervention type. Leave-one-out sensitivity analyses were conducted to assess the robustness of the pooled effect estimates. Small-study effects were evaluated using funnel plots and the Egger test, and the trim-and-fill method was applied to examine the robustness of the findings.

TSA was performed to control for potential random errors caused by repeated significance testing and sparse data. The required information size (RIS) was estimated using a 2-sided α of 5% and a statistical power of 80%, and monitoring boundaries were constructed using the O’Brien-Fleming method. TSA was conducted using the TSA software developed by the Copenhagen Trial Unit.

To compare the relative effectiveness of different DHIs, a frequentist network meta-analysis was performed for the primary outcome of viral suppression. Consistency between direct and indirect evidence was assessed using the node-splitting method. Relative treatment effects were summarized in a league table, and the surface under the cumulative ranking curve was used to estimate the ranking probability of each intervention. PI for comparisons between digital interventions and SOC were calculated using the Kenward-Roger adjustment.

All statistical analyses were conducted using R (version 4.5.3; R Core Team) and Stata (version 17; StataCorp LLC). Pairwise meta-analyses were performed using the *metafor* package, and network meta-analyses were conducted using the *netmeta* package.

## Results

### Study Search and Selection

**Figure 1.**
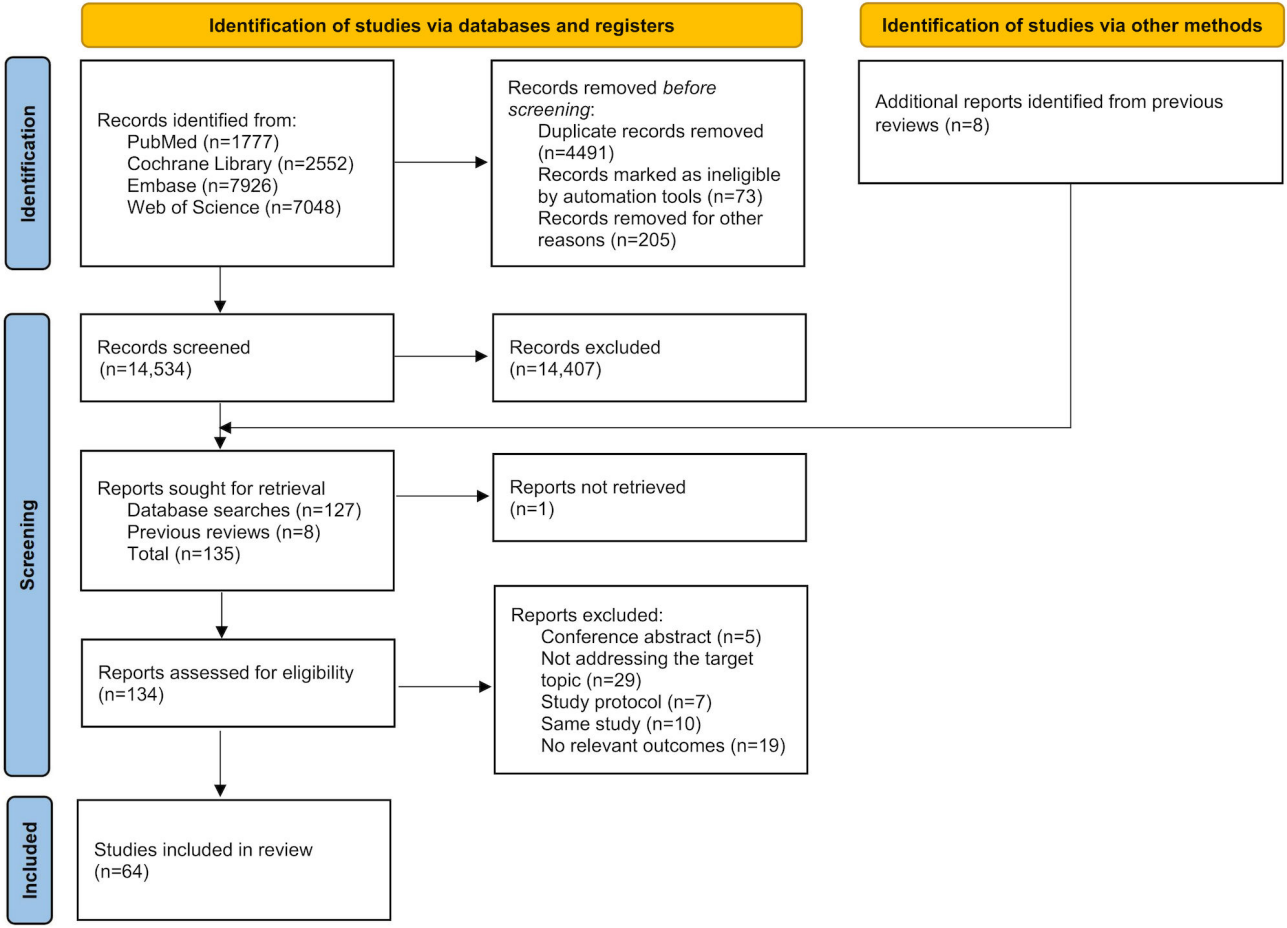
PRISMA (Preferred Reporting Items for Systematic Reviews and Meta-Analyses) 2020 flow diagram of study selection.

The study selection process is illustrated in Figure 1. The literature search was conducted initially and updated before submission using the same search strategy.The PRISMA flow diagram presents the combined results of both searches. After removal of duplicates, 14,407 records were excluded during title and abstract screening. The most common reasons for exclusion included nonrandomized study designs, studies not involving people living with HIV, interventions not meeting the definition of DHIs, and studies that did not report relevant ART-related outcomes. In addition, conference abstracts, commentaries, and reviews were excluded. After title and abstract screening, a total of 135 articles were sought for retrieval, and 134 articles were assessed for eligibility by full-text review after 1 article could not be retrieved. A total of 64 RCTs met the inclusion criteria [12,17-19,40-99]. All exclusions during screening were clearly based on predefined eligibility criteria, and no ambiguous cases requiring subjective judgment were identified.

### Study Characteristics

The main characteristics of the included studies are summarized in Table 1 and Table S1 in Multimedia Appendix 1. A total of 64 studies involving 22,286 participants were included [12,17-19,40-89,91-99]. In terms of intervention type, 27 studies evaluated SMS interventions [12,17-19,40,41,49,51,53,59,61,63-66,68,72-74,79,83,84,88,91,95,98], 10 assessed adherence monitoring devices [46,47,56,62,67,77,78,80,91,92], 8 evaluated mobile apps [54,55,57,58,71,87,93,94], 9 examined phone calls [48,52,69,70,76,85,86,89,90], 5 assessed multimedia education [45,81,82,96,97], and 9 used multiple digital interventions [18,42-44,50,53,60,75,78]. Follow-up duration ranged from 1 to 24 months. Most studies were conducted in the Americas and Africa, and 35 were carried out in low- and middle-income countries. The study populations primarily consisted of adults living with HIV, although several studies also included specific subgroups, such as men who have sex with men, adolescents living with HIV, and pregnant women living with HIV.

A total of 59 studies reported reach, with a median reach of 33.5% (IQR 21%‐42.9%). Uptake was reported in 37 studies, with a median of 85.7% (IQR 68.1%‐95.6%).

**Table 1.** Characteristics of included studies.

Study	Mode of delivery	Region	Study population	Follow-up (months)	Participants, n (intervention/control)	Age (y), mean (SD)		Sex (female), n (%)		Conclusion	Percentage reach, randomly assigned proportion (%)	Percentage uptake (%)	Feasibility
						Intervention	Control	Intervention	Control				
Abdulrahman et al [99]	SMS	Malaysia	Adults with HIV	6	242 (121/121)	32.1 (8.7)	34.7 (9.5)	14 (11.6)	14 (11.5)	Effective	18.1 (50)	96	Reported high adherence and effectiveness
Abiodun et al [12]	SMS	Nigeria	Adolescents living with HIV	12	209 (105/104)	16.6 (1.4)	16.7 (1.4)	55 (52.4)	53 (51)	Effective	41.7 (50.2)	83.4	Feasibility is based on effectiveness, response rate, and acceptability (95.3%)
Abuogi et al [98]	SMS	Kenya	Pregnant women living with HIV	12	1331 (668/663)	28.5 (5.7)	28.56 (5.5)	668 (100)	663 (100)	Ineffective for adherence and retention	41 (50.2)	—^a^	Feasibility is based on high adherence and fidelity
Aunon et al [95]	SMS	Kenya	Women living with HIV	6	119 (60/59)	33.6 (8.0)	34.2 (8.2)	60 (100)	59 (100)	Effective for short-term adherence	44.4 (50.4)	55	High acceptability (86.5%) and satisfaction
Boer et al [91]	SMS	Tanzania	Adults with HIV	12	166 (83/83)	39.6 (12)	41.2 (12)	0 (0)	0 (0)	Ineffective	61.5 (65.5)	—	Reported adherence and effectiveness
Christopoulos et al [19]	SMS	United States	Adults with HIV	12	230 (116/114)	44.5 (9)	43.3 (12)	13 (11.8)	17 (15.5)	Ineffective for viral suppression	20.4 (50.4)	97	High retention rate and satisfaction
da Costa et al [88]	SMS	Brazil	Women living with HIV	5	21 (8/13)	36.13 (9.14)	33.69 (5.34	13 (100)	8 (100)	Effective	15.3 (36)	100	Reported satisfaction and adherence
Davey et al [79]	SMS	Mozambique	Adults with HIV	12	826 (412/414)	38 (0.7)	37.3 (0.3)	253 (60.8)	242 (58.5)	Ineffective	34.3 (49.6)	—	High retention rate and high recruitment rate
Elul et al [84]	SMS	Mozambique	Adults with HIV	12	2004 (1237/767)	—	—	428 (34.6)	284 (37)	Effective	23.2 (61.7)	—	High retention rate and recruitment rate
Garofalo et al [83]	SMS	United States	Adults with HIV	6	105 (51/54)	24.1 (3.2)	24.1 (2.7)	10 (19.6)	8 (15.1)	Effective for adherence	29.7 (48.6)	89	High accessibility and 95% satisfaction
Ingersoll et al [72]	SMS	United States	Adults with HIV	3	63 (33/30)	42.1 (9.1)	42.7 (11.0)	13 (40.6)	10 (34.5)	Effective	7.6 (52.4)	68	High satisfaction
Kalichman et al [18]	SMS	United States	Adults with HIV	12	301 (150/151)	46.8 (9.0)	46.8 (10.0)	45 (30)	54 (35.8)	Effective	11.4 (50)	—	Reported adherence and effectiveness
Ketchaji et al [74]	SMS	Cameroon	Adults with HIV	6	92 (46/46)	—	—	—	—	Effective	47 (46.8)	68.5	High acceptability
Kiruthu-Kamamia et al [73]	SMS	Malawi	Adults with HIV	6	442 (214/228)	—	—	116 (54.2)	110 (48.2)	Ineffective	44.7 (66.3)	86.4	Reported adherence
Lester et al [17]	SMS	Kenya	Adults with HIV	12	538 (273/265)	36.7 (8.5)	36.6 (7.9)	94 (34.4)	95 (35.8)	Effective	41.6 (49.4)	—	High satisfaction and high acceptability
Linnemayr et al [68]	SMS	Uganda	Adults with HIV	12	332 (220/112)	18.4	18.2	96 (43.6)	55 (49.1)	Effective	NR (50)	—	Reported adherence and effectiveness
Lizárraga et al [66]	SMS	Peru	Adults with HIV	6	166 (82/84)	—	—	5 (6)	5 (6.1)	Ineffective	44.3 (50.5)	100	Reported satisfaction, adherence, and effectiveness
Maduka and Tobin-West [65]	SMS	Nigeria	Adults with HIV	4	104 (52/52)	—	—	26 (50)	33 (63.5)	Effective for retention	43 (49.9)	94	High retention rate and recruitment rate
Mbuagbaw et al [64]	SMS	Cameroon	Adults with HIV	6	200 (101/99)	41.3 (10.1)	39 (10.0)	69 (68.3)	78 (78.8)	Effective in the weekly text message subgroup	39.3 (67.5)	—	Fidelity, adherence, and retention rate
McNairy et al [63]	SMS	Eswatini	Adults with HIV	12	2197 (1096/1101)	32 (2.2)	30 (2.2)	657 (59.9)	637 (57.9)	Effective	17.7 (50)	97.9	High acceptability and 96% satisfaction
Pop-Eleches et al [61]	SMS	Kenya	Adults with HIV	12	428 (289/139)	35.64	35.65	189 (65.4)	92 (66.2)	Effective	12.5 (50.4)	41	Reported adherence and effectiveness
Ruan et al [59]	SMS	China	Adults with HIV	6	100 (50/50)	38.9 (9.8)	41.8 (9.8)	19 (38)	22 (44)	Effective	33.5 (77.8)	—	Reported retention rate
Simoni et al [49]	SMS	United States	Adults with HIV	9	224 (110/114)	—	—	—	—	Effective	—	—	High adherence rate; reported effectiveness
Steward et al [53]	SMS	South Africa	Adults with HIV	12	456 (289/167)	—	—	173 (59.9)	97 (58.1)	Effective	—	—	Reported retention rate
Tarantino et al [40]	SMS	Ghana	Adults with HIV	12	60 (30/30)	20.10 (1.9)	20.57 (2)	14 (46.7)	13 (43.3)	Effective	—	84.1	High adherence rate; reported effectiveness
Trinidad et al [41]	SMS	Mexican	Adults with HIV	6	80 (40/40)	—	—	2 (5)	3 (7.5)	Effective	42.8 (48.4)	—	High retention rate and satisfaction
van der Kop et al [51]	SMS	Kenya	Adults with HIV	12	700 (349/351)	34 (10.1)	33.5 (9.4)	206 (59)	213 (60.7)	Ineffective	32.7 (49.9)	56	Reported retention rate and satisfaction
Boer et al [91]	Adherence monitor	Tanzania	Adults with HIV	12	166 (83/83)	42.8 (12)	41.2 (12)	—	—	Ineffective	61.5 (65.5)	—	Reported adherence and effectiveness
Byonanebye et al [92]	Adherence monitor	Uganda	Adults with HIV	12	600 (300/300)	—	—	210 (70)	203 (67.7)	Ineffective	27.8 (50)	52.8	Reported adherence and effectiveness
Ellsworth et al [80]	Adherence monitor	United States	Adults with HIV	3	63 (30/33)	52 (6.2)	47.6 (7.7)	—	—	Ineffective	44.8 (47.6)	96	Reported adherence and effectiveness
Haberer et al [78]	Adherence monitor	Uganda	Adults with HIV	9	41 (20/21)	—	—	7 (36.8)	18 (85.7)	Effective for adherence	21 (66.1)	63	High adherence rate and reported effectiveness
Knox et al [77]	Adherence monitor	United States	Adults with HIV	12	114 (77/37)	—	—	30 (39)	18 (48.6)	Effective	42.8 (67.5)	98	High usage, high retention rate, and high adherence
Liu et al [67]	Adherence monitor	United States	Adults with HIV	3	112 (54/58)	46.7 (11.1)	45.7 (12.4)	5 (10.2)	5 (10.4)	Effective for adherence	39.7 (48.2)	—	High satisfaction
Moore et al [62]	Adherence monitor	United States	Adults with HIV	1	50 (25/25)	48.4 (9.2)	45.9 (10.2)	3 (11.5)	4 (16)	Ineffective for adherence	40.3 (50)	92.3	Reported adherence
Orrell et al [46]	Adherence monitor	South Africa	Adults with HIV	12	230 (115/115)	34.6 (9.2)	34.3 (9)	73 (63.5)	77 (67)	Ineffective	36.1 (50)	52.4	Reported retention rate, adherence, and effectiveness
Sabin et al [47]	Adherence monitor	China	Adults with HIV	9	119 (63/56)	36.9 (11.1)	38.4 (9.6)	21 (33.3)	22 (39.3)	Effective for adherence	38 (52.9)	91.6	Reported adherence and effectiveness
Sabin et al [56]	Adherence monitor	Uganda	Pregnant women living with HIV	3	133 (69/64)	25.6 (6.8)	25.2 (4.6)	69 (100)	64 (100)	Ineffective	41.8 (51.9)	—	Reported retention rate
Ayer et al [94]	App	Nepal	Adults with HIV	6	468 (234/234)	37.3 (10.7)	36.5 (9.9)	109 (46.6)	99 (42.3)	Effective for adherence	45 (50)	91	91% satisfaction and adherence
Belzer et al [93]	App	United States	Adults with HIV	12	37 (19/18)	19.84 (2.52)	21.06 (2.53)	8 (42.1)	6 (33.3)	Effective	47.5 (51.4)	—	Adherence and effectiveness
DeFulio et al [87]	App	United States	Adults with HIV	1	50 (25/25)	52.4 (10.7)	54 (7.8)	17 (68)	9 (36)	Effective	39.7 (50)	81	High acceptability and satisfaction
Jiao et al [71]	App	China	MSM^b^ living with HIV	3	576 (288/288)	—	—	0 (0)	0 (0)	Effective for adherence	48.6 (50)	—	Reported adherence and effectiveness
Ruel et al [58]	App	Kenya	Adults with HIV	24	1549 (785/764)	—	—	643 (81.9)	605 (79.2)	Effective	39.5 (50.7)	—	High satisfaction, retention rate, and effectiveness
Saberi et al [57]	App	United States	Adults with HIV	4	50 (25/25)	25.8 (2.7)	24.7 (3.2)	3 (12)	3 (13.6)	Ineffective for adherence and viral suppression	NA (50)	76	High acceptability and high satisfaction
Sherman et al [55]	App	United States	Adults with HIV	6	94 (45/49)	37.5 (11.7)	40.7 (10.8)	17 (37.8)	19 (38.8)	Effective for retention	32.6 (47.9)	—	High retention rate; reported adherence and effectiveness
Shet et al [54]	App	India	Adults with HIV	24	631 (315/316)	—	—	136 (43.2)	137 (43.4)	Ineffective	27.6 (49.9)	97	High fidelity
Claborn et al [89]	Phone call	United States	Adults with HIV	1	97 (47/50)	43.7 (10.19)	42 (9.47)	9 (19.1)	7 (14.3)	Effective for adherence	10.7 (48.5)	—	Adherence and 85.1% satisfaction
DiPrete et al [86]	Phone call	United States	Adults with HIV	6	381 (195/186)	42.6 (10.5)	41.9 (11.2)	48 (24.6)	36 (19.4)	Ineffective	10.8 (51.2)	79	Reported adherence and effectiveness
Dulli et al [85]	Phone call	Nigeria	Adults with HIV	12	349 (177/172)	21.3 (2.3)	21.0 (2.3)	151 (85.3)	155 (90.1)	Ineffective	46.7 (50.7)	94.4	High acceptability
Huang et al [76]	Phone call	China	Adults with HIV	3	196 (98/98)	—	—	—	—	Ineffective	33.1 (50)	81.7	Reported retention rate and effectiveness
Kalichman et al [70]	Phone call	United States	Adults with HIV	12	157 (77/80)	43.3 (12.8)	41.1 (11.9)	24 (31.2)	26 (32.5)	Ineffective	32.1 (49)	81	Reported retention rate and effectiveness
Kim et al [69]	Phone call	Malawi	Women living with HIV	1	298 (142/156)	27.5 (5.7)	27.3 (9.1)	—	—	Ineffective	Not available (47.7)	100	91.1% satisfaction and high acceptability
Sarna et al [48]	Phone call	Kenya	Pregnant women living with HIV	12	404 (207/197)	—	—	207 (100)	197 (100)	Effective	9.5 (51.2)	63	Reported retention rate
Satyanarayana et al [90]	Phone call	India	Women living with HIV	24	120 (60/60)	37.1 (8.3)	38.2 (8.8)	60 (100)	60 (100)	Effective	41.5 (50)	—	Reported retention rate and effectiveness
Uzma et al [52]	Phone call	Pakistan	Adults with HIV	2.5	68 (34/34)	—	—	12 (31.6)	8 (21.1)	Effective	Not available (50)	—	Reported adherence and effectiveness
Amico et al [97]	Multimedia education	United States	Adults with HIV	12	88 (43/45)	21.83 (1.88)	21.71 (2.54)	22 (51.2)	26 (57.8)	Effective for adherence	16 (49)	100	High adherence; reported effectiveness
Andrade-Romo et al [96]	Multimedia education	Mexico	MSM living with HIV	10	151 (74/77)	30	31	0 (0)	0 (0)	Ineffective	22.5 (49)	—	Reported adherence
Fayorsey et al [81]	Multimedia education	Kenya	Pregnant women living with HIV	6	340 (170/170)	26.4 (6.7)	25.5 (6.7)	170 (100)	170 (100)	Effective for retention	47.1 (50)	—	Reported retention rate and effectiveness
Guo et al [82]	Multimedia education	China	Adults with HIV	3	62 (31/31)	29.2 (6.5)	27.4 (5.7)	5 (16.1)	1 (3.2)	Ineffective	Not reported (50)	85	High acceptability and satisfaction
Lewis et al [45]	Multimedia education	United States	Adults with HIV	12	799 (397/402)	—	—	94 (23.7)	95 (23.7)	Effective for retention	28 (49.7)	—	Reported retention rate and effectiveness
Haberer et al [78]	Multiple digital interventions	Uganda	Adults with HIV	9	42 (21/21)	—	—	15 (71.4)	18 (85.7)	Effective for adherence	21 (66.1)	63	High adherence rate; reported effectiveness
Horvath et al [75]	Multiple digital interventions	United States	MSM living with HIV	11	410 (208/202)	40.1 (10.8)	38.1 (10.6)	0 (0)	0 (0)	Effective	—	—	Reported effectiveness
Kalichman et al [18]	Multiple digital interventions	United States	Adults with HIV	12	299 (150/149)	47 (9.1)	47.8 (9.9)	38 (25.3)	35 (23.5)	Effective	11.4 (50)	—	Reported adherence and effectiveness
Mimiaga et al [44]	Multiple digital interventions	United States	Adults with HIV	12	123 (63/60)	25.1 (2.9)	25.8 (2.9)	8 (12.7)	7 (11.7)	Effective	18 (51.2)	54.2	Reported retention rate and effectiveness
Naggirinya et al [43]	Multiple digital interventions	Uganda	Adults with HIV	12	206 (103/103)	22.5 (1.9)	22.3 (2.3)	81 (78.6)	86 (83.5)	Effective	70.3 (50)	—	Reported retention rate and effectiveness
Ramsey et al [60]	Multiple digital interventions	United States	Adults with HIV	12	53 (27/26)	44.9 (14.1)	48.7 (10.2)	9 (33.3)	6 (23.1)	The pattern of results was consistent with better adherence in the intervention	15.7 (50.9)	86.5	High acceptability and high satisfaction
Schnall et al [42]	Multiple digital interventions	United States	Adults with HIV	6	300 (150/150)	46.8 (11.4)	49.4 (11.9)	66 (47.1)	68 (48.6)	Effective	40.6 (50)	75.8	Reported effectiveness
Steward et al [53]	Multiple digital interventions	South Africa	Adults with HIV	12	463 (296/167)	—	—	190 (64.2)	97 (58.1)	Effective	33.5 (77.8)	—	Reported retention rate
Whiteley et al [50]	Multiple digital interventions	United States	Adults with HIV	4	61 (32/29)	22.5 (2.5)	22.3 (2.5)	7 (21.9)	6 (20.7)	Effective	48.5 (52.5)	—	Reported effectiveness

a Not available.

b MSM: men who have sex with men.

### Risk of Bias

**Figure 2.**
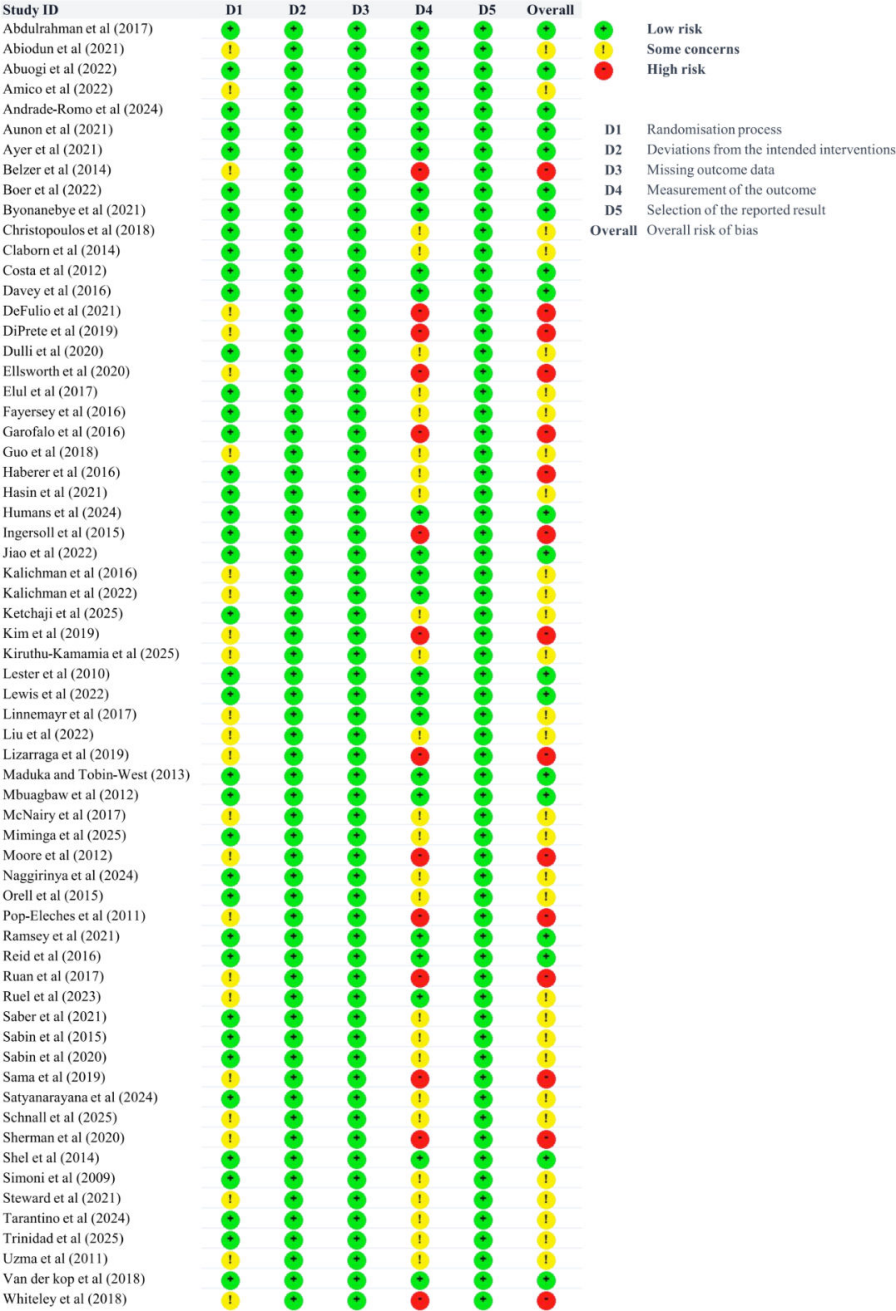
Risk of bias across effect estimates at Postintervention and follow-up assessments based on the Cochrane risk of bias 2 tool for randomized trials [12,17-19,40-99].

The risk-of-bias assessment showed that 19 studies were rated as having low risk of bias [17,45,51,54,60,64,65,71,75,76,79,88,91,92,94-96,98,99], 30 raised some concerns [12,18,19,40-44,46,47,49,52,53,56-58,63,67,68,70,73,74,77,81,82,84,85,89,90,97], and 15 were judged to be at high risk of bias [48,50,55,59,61,62,66,69,72,78,80,83,86,87,93]. In 25 studies [18,19,41,43,44,48-50,52,54,56,57,60,62-64,72-74,77,78,80,88,95,98], although randomization was reported, the allocation concealment procedures were not clearly described, which may have introduced bias in the randomization process. In addition, 25 studies were considered to have potential bias in outcome measurement [19,40-44,46,47,49,52,53,56,57,63,67,73,74,77,78,81,82,84,85,89,90]. Detailed results of the risk-of-bias assessment are presented in Figure 2.

### Meta-Analysis: Viral Suppression and CD4^+^ Cell Counts

**Figure 3.**
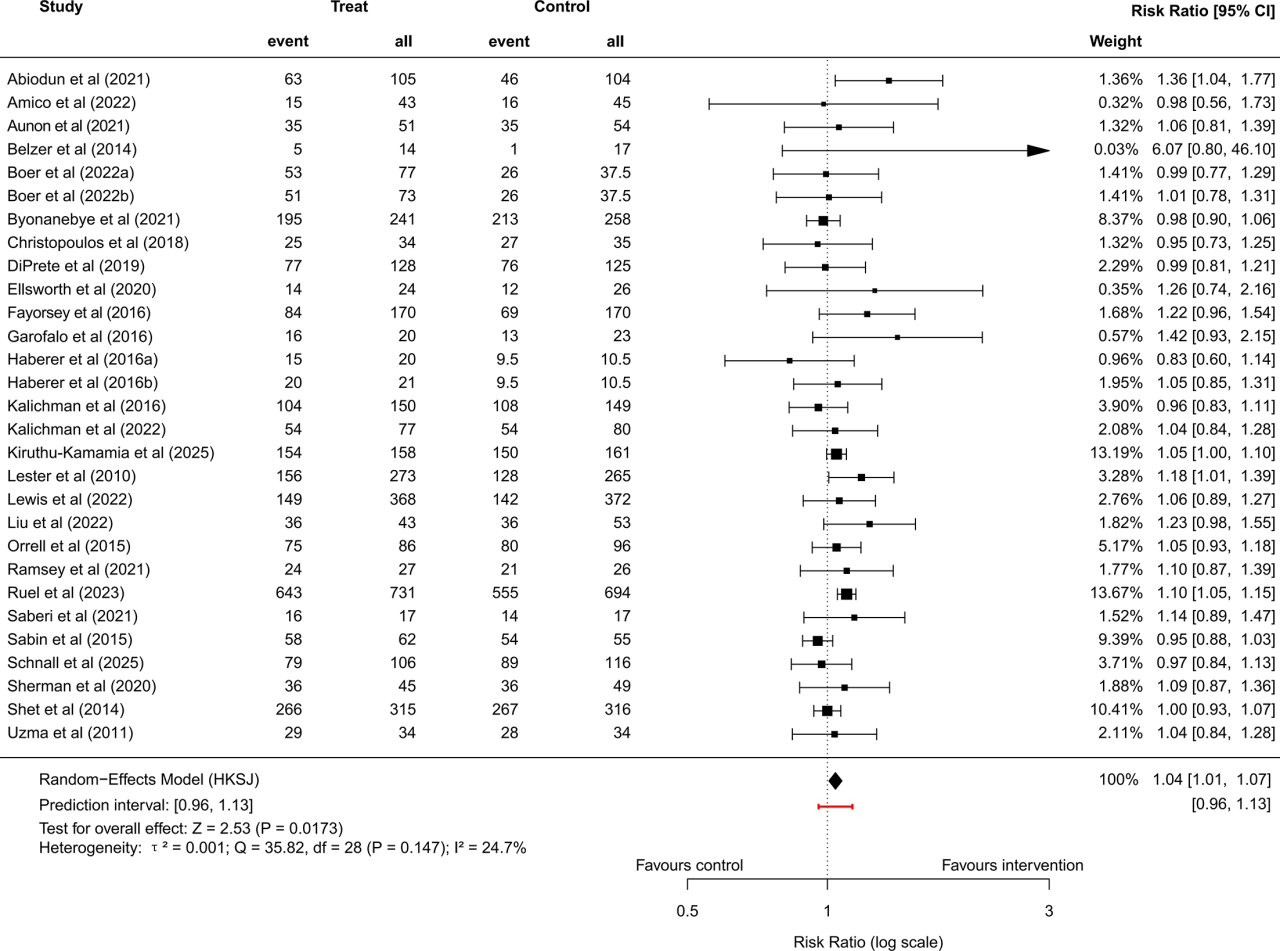
Meta-analysis of viral suppression outcomes comparing intervention and control groups among people living with HIV. Boer et al (2022a) and Boer et al (2022b), as well as Haberer et al (2016a) and Haberer et al (2016b), refer to different intervention arms from the same study [12,17-19,42,45-47,52,54,55,57,58,60,67,70,73,78,80,81,83,86,91-93,95,97].

A total of 27 studies reported viral suppression outcomes. Compared with SOC, DHIs were associated with a modest improvement in viral suppression (RR 1.04, 95% CI 1.01‐1.07). Heterogeneity was low (*τ*=0.032; *τ*²=0.001; Q=36.38; *I*²=24.4%; *P*=.13). However, the PI was relatively wide and crossed the null (95% PI 0.96‐1.13), indicating substantial variability in the potential effects across settings (Figure 3); however, the certainty of evidence was rated as very low (Table 2). TSA indicated that the cumulative Z-curve crossed both the conventional boundary and the TSA monitoring boundary before reaching the RIS and remained above the boundary thereafter, suggesting that the evidence was statistically robust (Figure S2.1 in Multimedia Appendix 1). Subgroup analysis by intervention type showed that, compared with SOC, multiple digital interventions were significantly associated with improved viral suppression (RR 1.09, 95% CI 1.01‐1.17; *I*²=0; *τ*=0; *τ*²=0; 95% PI 1.01‐1.17; *P*=.87; Figure S3.1 in Multimedia Appendix 1). The 95% PI suggests a high likelihood that the intervention would confer at least a small beneficial effect in future similar study settings. The certainty of evidence was rated as low (Table 2). In contrast, other intervention types did not demonstrate statistically significant effects (Figure S3.1 in Multimedia Appendix 1).

**Table 2.** GRADE (Grading of Recommendations Assessment, Development, and Evaluation) ratings at postintervention assessments.

	Certainty assessment							Summary of findings			
	Participants (studies) follow-up	Risk of bias	Inconsistency	Indirectness	Imprecision	Publication bias	Overall certainty of evidence	Study event rates		Relative effect, RR^a^ (95% CI)	Anticipated absolute effects (risk with standard care)
								With standard care	With DHIs^b^		
Virus suppression											
Total	6949 (27 RCTs^c^)	Serious^d^	Very serious^e,f^	Not serious	Not serious	None	⨁◯◯◯ Very low^d,e,f^	2547/3436 (74.1%)	2341/3513 (66.6%)	1.04 (1.01-1.07)	2547/3436 (74.1%)
SMS	1661 (7 RCTs)	Serious^d^	Serious^f^	Not serious	Serious^g^	None	⨁◯◯◯ Very low^d,f,g^	531/831 (63.9%)	579/830 (69.8%)	1.08 (0.97-1.20)	531/831 (63.9%)
Adherence monitor	638 (6 RCTs)	Serious^d^	Very serious^e,f^	Not serious	Serious^g^	None	⨁◯◯◯ Very low^d,e,f,g^	253/326 (77.6%)	251/312 (80.4%)	1.01 (0.90-1.13)	253/326 (77.6%)
App	2683 (5 RCTs)	Serious^d^	Serious^f^	Not serious	Serious^g^	None	⨁◯◯◯ Very low^d,f,g^	1085/1334 (81.3%)	1156/1349 (85.7%)	1.04 (0.97-1.12)	1085/1334 (81.3%)
Phone call	509 (4 RCTs)	Serious^d^	Serious^f^	Not serious	Serious^g^	None	⨁◯◯◯ Very low^d,f,g^	159/256 (62.1%)	165/253 (65.2%)	1.03 (0.84-1.25)	159/256 (62.1%)
Multiple digital interventions	1263 (5 RCTs)	Serious^d^	Serious^f^	Not serious	Not serious	None	⨁⨁◯◯ Low^d,f^	267/634 (42.1%)	292/629 (46.4%)	1.09 (1.01-1.17)	267/634 (42.1%)
Objective adherence											
Total	2742 (10 RCTs)	Serious^d^	Serious^f^	Not serious	Serious^g^	Publication bias strongly suspected	⨁◯◯◯ Very low^d,f,g,h^	622/1253 (49.6%)	762/1489 (51.2%)	1.19 (0.95-1.50)	622/1253 (49.6%)
SMS	1999 (7 RCTs)	Serious^d^	Serious^f^	Not serious	Serious^g^	None	⨁◯◯◯ Very low^d,f,g^	347/880 (39.4%)	491/1119 (43.9%)	1.22 (0.91-1.65)	347/880 (39.4%)
App	743 (3 RCTs)	Serious^d^	Serious^f^	Not serious	Serious^g^	None	⨁◯◯◯ Very low^d,f,g^	275/373 (73.7%)	271/370 (73.2%)	1.23 (0.37-4.04)	275/373 (73.7%)
Subjective adherence											
Total	5323 (23 RCTs)	Serious^d^	Serious^f^	Not serious	Not serious	None	⨁⨁◯◯ Low^d,f^	1753/2636 (66.5%)	1999/2687 (74.4%)	1.13 (1.04-1.23)	1753/2636 (66.5%)
SMS	3214 (11 RCTs)	Serious^d^	Serious^f^	Not serious	Not serious	None	⨁⨁◯◯ Low^d,f^	1003/1609 (62.3%)	1154/1605 (71.9%)	1.23 (1.09-1.38)	1003/1609 (62.3%)
Adherence monitor	100 (2 RCTs)	Serious^d^	Serious^f^	Not serious	Serious^g^	None	⨁◯◯◯ Very low^d,f,g^	17/51 (33.3%)	21/49 (42.9%)	1.29 (0.37-4.46)	17/51 (33.3%)
App	610 (2 RCTs)	Serious^d^	Serious^f^	Not serious	Serious^g^	None	⨁◯◯◯ Very low^d,f,g^	210/305 (68.9%)	247/305 (81%)	1.17 (0.78-1.75)	210/305 (68.9%)
Phone call	567 (3 RCTs)	Serious^d^	Serious^f^	Not serious	Serious^g^	none	⨁◯◯◯ Very low^d,f,g^	206/285 (72.3%)	233/282 (82.6%)	1.11 (0.84-1.46)	206/285 (72.3%)
Multimedia education	395 (2 RCTs)	Serious^d^	Serious^f^	Not serious	Serious^g^	None	⨁◯◯◯ Very low^d,f,g^	160/206 (77.7%)	123/189 (65.1%)	0.83 (0.61-1.13)	160/206 (77.7%)
Multiple digital interventions	437 (3 RCTs)	Serious^d^	Serious^f^	Not serious	Serious^g^	None	⨁◯◯◯ Very low^d,f,g^	157/180 (87.2%)	221/257 (86%)	0.98 (0.94-1.03)	157/180 (87.2%)
Retention											
Total	16,221 (33 RCTs)	Serious^d^	Very serious^e,f^	Not serious	Not serious	None	⨁◯◯◯ Very low^d,e,f^	5115/7570 (67.6%)	6378/8651 (73.7%)	1.06 (1.01-1.12)	5115/7570 (67.6%)
SMS	9943 (15 RCTs)	Serious^d^	Very serious^e,f^	Not serious	Serious^g^	None	⨁◯◯◯ Very low^d,e,f,g^	2826/4454 (63.4%)	3850/5489 (70.1%)	1.09 (0.99-1.19)	2826/4454 (63.4%)
Adherence monitor	475 (3 RCTs)	Serious^d^	Serious^f^	Not serious	Serious^g^	None	⨁◯◯◯ Very low^d,f,g^	164/216 (75.9%)	187/259 (72.2%)	0.94 (0.74-1.19)	164/216 (75.9%)
App	2159 (3 RCTs)	Serious^d^	Serious^f^	Not serious	Serious^g^	None	⨁◯◯◯ Very low^d,f,g^	767/1073 (71.5%)	936/1086 (86.2%)	1.17 (0.87-1.57)	767/1073 (71.5%)
Phone call	1475 (6 RCTs)	Serious^d^	Serious^f^	Not serious	Serious^g^	None	⨁◯◯◯ Very low^d,f,g^	573/743 (77.1%)	605/732 (82.7%)	1.06 (0.93-1.21)	573/743 (77.1%)
Multimedia education	1139 (2 RCTs)	Serious^d^	Serious^f^	Not serious	Serious^g^	None	⨁◯◯◯ Very low^d,f,g^	369/572 (64.5%)	395/567 (69.7%)	1.09 (0.69-1.70)	369/572 (64.5%)
Multiple digital interventions	1030 (4 RCTs)	Serious^d^	Serious^f^	Not serious	Serious^g^	None	⨁◯◯◯ Very low^d,f,g^	416/512 (81.3%)	405/518 (78.2%)	0.98 (0.80-1.19)	416/512 (81.3%)
CD4+ cell counts	1342 (11 RCTs)	Serious^d^	Very serious^e,f^	Not serious	Serious^g^	None	⨁◯◯◯ Very low^d,e,f,g^	667	675	—^i^	667

a RR: risk ratio.

b DHI: digital health intervention.

c RCT: randomized controlled trial.

d Downgraded by one level for risk of bias because several included studies were judged to be at high risk of bias.

e Downgraded by one level for inconsistency because of substantial variability in point estimates across studies or minimal to no overlap of CIs.

f Downgraded by one level due to the prediction interval crossing the line of no effect.

g Downgraded by one level due to the 95% CI crossing the line of no effect.

h Downgraded by one level for small-study effects because the funnel plot showed asymmetry, Egger test was significant (*P*<.05), and the trim-and-fill analysis suggested that the pooled estimate may be affected by missing or small studies.

i Not available.

A total of 11 studies reported end-of-study CD4^+^ cell counts. The pooled analysis showed no significant difference between DHIs and SOC (Figure S4 in Multimedia Appendix 1). The cumulative Z-curve crossed the conventional boundary but did not reach the RIS, indicating that the current evidence remains inconclusive (Figure S2.2 in Multimedia Appendix 1).

### Treatment Adherence

**Figure 4.**
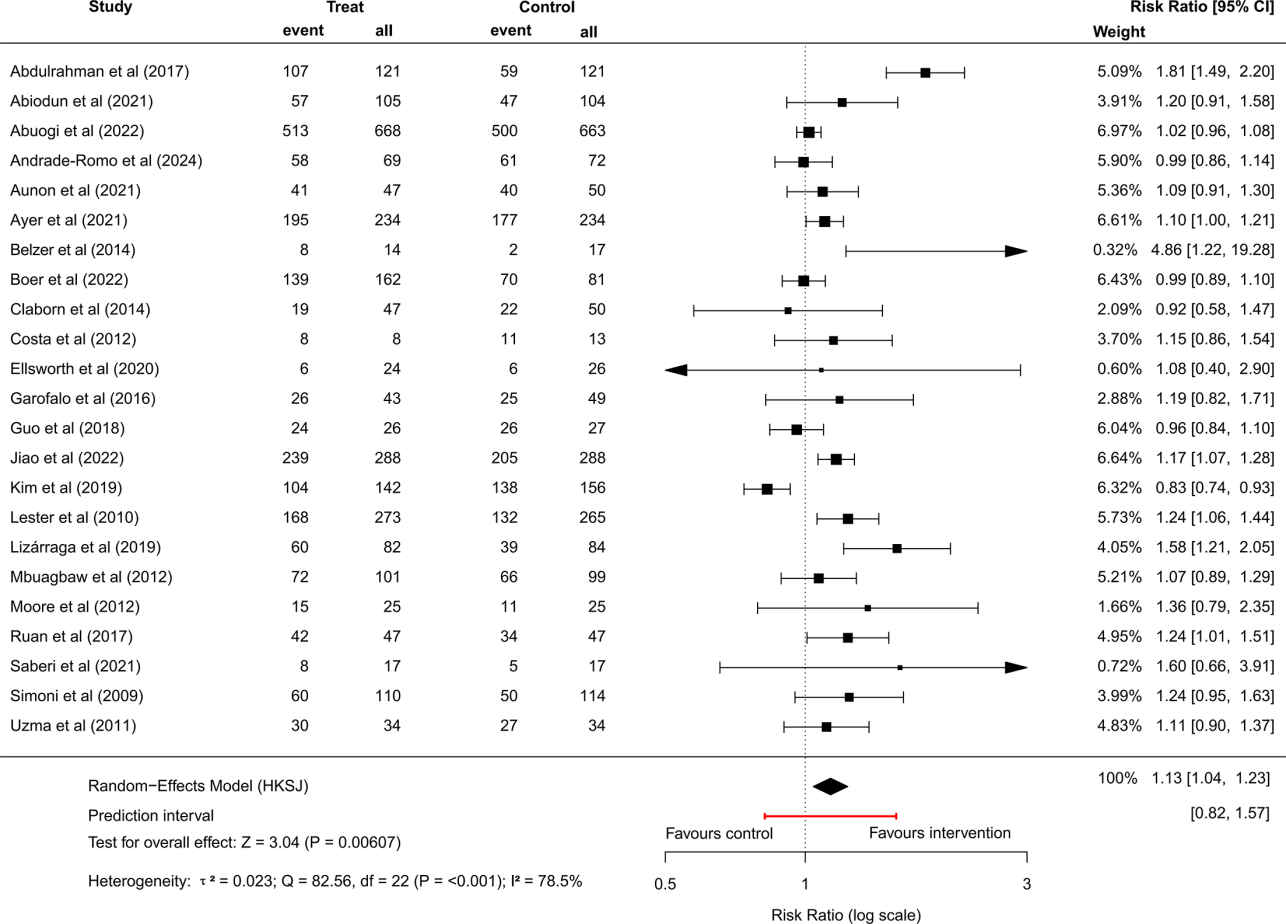
Meta-analysis of subjective adherence comparing intervention and control groups among people living with HIV [12,17,49,52,57,59,62,64,66,69,71,80,82,83,88,89,91,93-96,98,99].

A total of 23 studies reported subjective adherence outcomes. The pooled results showed that, compared with SOC, DHIs significantly improved subjective adherence, with low certainty of evidence (RR 1.13, 95% CI 1.04‐1.23), and the PI (0.82‐1.57) suggests considerable variability in future outcomes. Significant heterogeneity was found (*I*^2^=78.5%, *P*<.001), and the heterogeneity was substantial (*τ*=0.152; τ^2^=0.023; Figure 4; Table 2). TSA indicated that the cumulative Z-curve crossed both the conventional boundary and the TSA monitoring boundary before reaching the RIS and remained beyond the boundary thereafter, suggesting that the evidence was sufficient and statistically robust (Figure S2.3 in Multimedia Appendix 1). Subgroup analyses showed that SMS was significantly associated with improvements in subjective adherence (RR 1.23, 95% CI 1.09‐1.38; *I*^2^=73.7%; *τ*=0.148; *τ*^2^=0.022; PI 0.86‐1.75; *P*<.001), whereas other intervention types did not demonstrate statistically significant effects (Figure S3.2 in Multimedia Appendix 1).

A total of 10 studies reported objective adherence outcomes (Figure S5 in Multimedia Appendix 1). The pooled meta-analysis showed no statistically significant difference between DHIs and SOC, with very low certainty of evidence. Subgroup analyses indicated no statistically significant differences among different types of interventions (Figure S3.3 in Multimedia Appendix 1)

### Retention

A total of 33 studies reported retention outcomes. The pooled meta-analysis indicated that DHIs were associated with higher retention compared with SOC (RR 1.06, 95% CI 1.01‐1.12). Substantial heterogeneity was observed (*τ*=0.130; *τ*²=0.017;* I*²=88.3%; *P*<.001). However, the certainty of evidence was rated as very low, and the PI (0.81‐1.39) crossed the null, suggesting considerable variability in the potential effects across settings (Figures S3.4 and S6 in Multimedia Appendix 1). TSA showed that the cumulative Z-curve remained within the monitoring boundaries and entered the futility area before reaching the RIS, suggesting that the observed positive effect may not be robust (Figure S2.5 in Multimedia Appendix 1). Subgroup analyses indicated no statistically significant differences among different types of interventions (Figure S3.4 in Multimedia Appendix 1).

### Sensitivity Analysis and Publication Bias

Leave-one-out sensitivity analyses indicated that the pooled estimates for viral suppression, subjective adherence, and retention were relatively robust, whereas the results for objective adherence and CD4^+^ cell counts were less stable (Table S2 in Multimedia Appendix 1). Funnel plots for viral suppression, retention, and subjective adherence appeared largely symmetrical (Figure S7 in Multimedia Appendix 1). Egger test suggested potential small-study effects for objective adherence and CD4^+^ cell count outcomes, whereas no such effects were detected for the other outcomes. Trim-and-fill analyses showed that the pooled effect estimate for CD4^+^ cell counts remained stable after adjustment, suggesting a limited impact of publication bias. In contrast, the pooled estimate for objective adherence changed substantially after adjustment, indicating the possible presence of publication bias and reduced robustness of this outcome.

### Network Meta-Analysis for Viral Suppression

**Figure 5.**
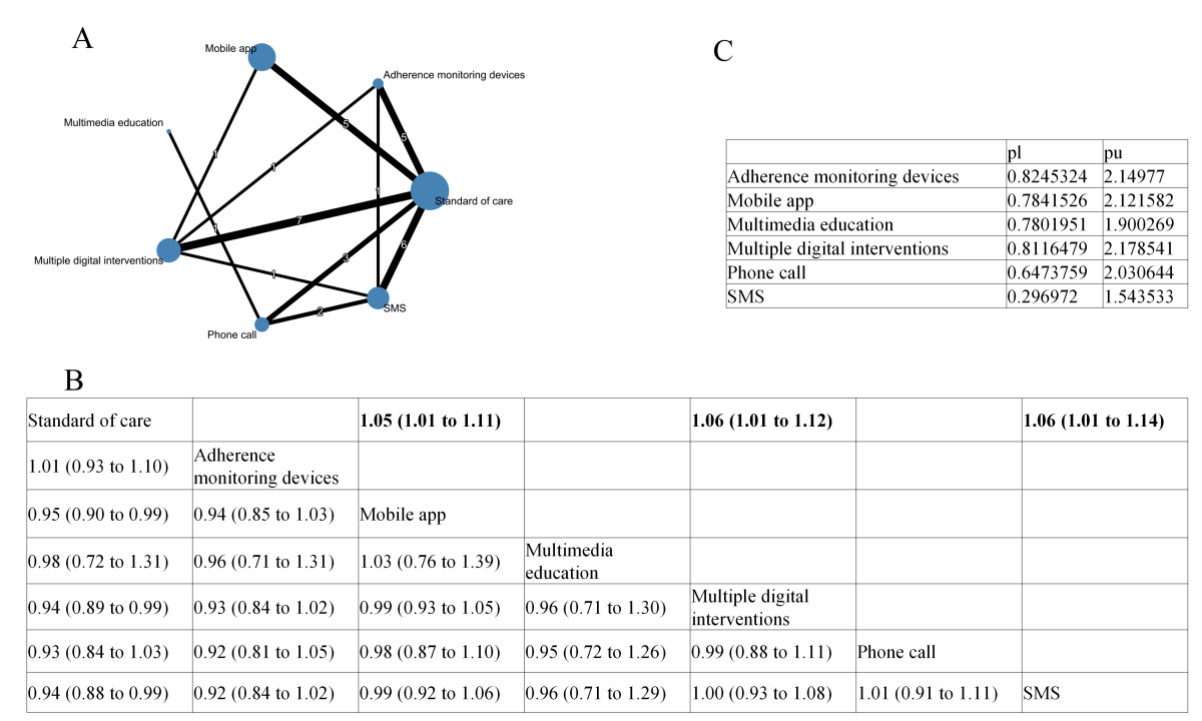
Network meta-analysis of viral suppression. (A) Network plot showing the direct comparisons among intervention categories; node size reflects the amount of evidence for each intervention, and edge thickness reflects the number of direct comparisons. (B) League table presenting the relative treatment effects between interventions estimated from the network shown in part A. (C) Prediction intervals for each intervention compared with standard of care, based on the same network meta-analysis.

A total of 27 studies were included in the network meta-analysis of viral suppression (Figure 5). The league table showed that mobile apps, multiple digital interventions, and SMS text were significantly more effective than SOC in improving viral suppression. No statistically significant differences were observed between the other intervention types. According to the SUCRA rankings, the interventions were ordered from most to least effective as follows: phone call (69.6%), SMS text (69.1%), multiple digital interventions (68.9%), mobile apps (59.1%), multimedia education (45.8%), SOC (19.6%), and adherence monitoring devices (18.0%). However, the PIs for all comparisons versus SOC crossed the line of no effect (Figure 5).

## Discussion

### Principal Findings

This systematic review offers an updated and comprehensive synthesis of randomized evidence on DHIs for improving ART outcomes in people living with HIV, addressing important gaps in previous studies. Overall, DHIs were associated with modest improvements in adherence, viral suppression, and retention in care, while no clear effect was observed for CD4^+^ cell counts. Importantly, PIs consistently crossed the null, highlighting substantial variability in effects across settings. These findings indicate that the benefits of DHIs are neither uniform nor guaranteed, and their effectiveness likely depends on contextual and implementation factors.

Previous systematic reviews have primarily focused on the impact of DHIs on adherence, with recent meta-analyses reporting that DHIs significantly improve ART adherence [20-22]. In contrast, our study distinguished between subjective and objective adherence measures and identified notable differences between them. Improvements were observed in subjective adherence, which may reflect the behavioral mechanisms underlying interventions such as SMS reminders that provide timely prompts and reinforcement [100,101]. Although TSA suggested that the cumulative evidence reached statistical sufficiency, the wide PIs indicate considerable variability across implementation contexts. This finding is consistent with behavioral theory, where intervention effects are influenced not only by the intervention itself but also by individual and contextual factors [102,103]. In contrast, no statistically significant effect was observed for objective adherence, and the available evidence remains insufficient. In addition, we identified evidence of small-study effects, an issue not commonly reported in previous reviews. Sensitivity analyses suggested that the results were not robust, and trim-and-fill analyses further indicated the potential presence of such effects. These findings suggest that, when assessed using more stringent objective measures, the effectiveness of DHIs remains uncertain and warrants cautious interpretation.

Viral suppression is one of the most important clinical outcomes in HIV management [5]. In this systematic review, DHIs showed a trend toward improving viral suppression. Although TSA indicated that the cumulative evidence may be statistically sufficient, the certainty of evidence was rated as very low using GRADE, and the wide PIs further support the overall interpretation that, while potential benefits exist, their reliability and generalizability remain uncertain. Moreover, viral suppression is influenced not only by adherence but also by biological and measurement-related factors [104,105], which may contribute to variability in viral load assessments and partly explain why improvements in adherence do not consistently translate into virologic benefits across studies [104]. Contrary to our expectations, very low certainty evidence indicated no significant effect of DHIs on CD4^+^ cell counts. This may be explained by the fact that CD4^+^ recovery is a relatively slow and biologically complex process, primarily determined by ART efficacy, baseline immune status, and treatment duration, rather than short-term behavioral interventions alone [105,106]. In addition, compared with adherence or viral suppression, CD4^+^ counts are less sensitive to short-term changes and typically require longer follow-up to detect clinically meaningful differences [105]. Variations in assessment timing, laboratory methods, and baseline CD4^+^ levels across studies may further increase heterogeneity and reduce the ability to detect consistent effects [106,107]. Therefore, although DHIs may improve adherence, the translation of behavioral changes into immunological recovery is neither immediate nor linear, which may attenuate observable effects on CD4^+^ outcomes [105].

DHIs were also associated with a modest improvement in retention; however, the certainty of evidence was very low. The wide PIs and TSA findings suggest that this effect is not robust. Retention in care is influenced by multiple structural and social factors, including transportation barriers, stigma, health care accessibility, financial constraints, and psychosocial support [108-112]. DHIs may address some of these barriers but are unlikely to fully overcome them when implemented in isolation, which may explain the limited magnitude and uncertainty of the observed effect [109].

Substantial heterogeneity was observed across included studies. One likely source is the broad inclusion of diverse DHI types, ranging from simple interventions such as SMS text reminders to more complex, multicomponent strategies [12,19,42,75,97,99]. Although subgroup analyses were conducted by intervention type, significant heterogeneity persisted within subgroups. The findings suggested that multicomponent DHIs were associated with improvements in viral suppression, whereas SMS-based interventions were linked to improvements in subjective adherence. This pattern may reflect differences in mechanisms of action—SMS interventions are simple, scalable, and effective in supporting medication-taking behavior [12], while multicomponent interventions address multiple aspects of care, including reminders, monitoring, education, and patient-provider communication, and may therefore be more likely to influence complex clinical outcomes [44]. However, tests for subgroup differences were not statistically significant, and these findings should not be interpreted as evidence of superiority. Importantly, comparisons across trials do not reflect random allocation between different DHI types, limiting causal inference. Observed subgroup effects may instead reflect differences in populations, intervention intensity, comparators, or duration. Furthermore, the wide PIs for all major outcomes, consistently crossing the null, indicate that the effectiveness of DHIs in future settings may range from no benefit or even harm to meaningful improvement. This underscores that the same intervention may not be equally effective for all patients, and clinical decision-making should consider individual patient characteristics. Future research should aim to identify which DHI strategies are most effective for specific populations.

Network meta-analysis of viral suppression yielded results that were not fully consistent with those of the pairwise meta-analysis. While the network analysis suggested that SMS and mobile app interventions were superior to standard care, these differences were not consistently observed in pairwise comparisons. This discrepancy likely reflects differences in analytical frameworks, as pairwise meta-analysis relies solely on direct comparisons [31], whereas network meta-analysis integrates both direct and indirect evidence [31]. In the presence of limited head-to-head trials, sparse networks, and substantial heterogeneity, the stability of network estimates may be reduced [113]. Moreover, PI for all comparisons crossed the line of no effect, indicating that the ranking of interventions should not be interpreted as evidence of a clear and robust advantage of any specific DHI type.

### Comparison With Previous Systematic Reviews

Our findings are broadly consistent with previous meta-analyses showing that DHIs are associated with improvements in adherence and viral suppression [20-23], and we additionally observed a potential benefit in retention in care. Compared with earlier studies, this systematic review has several strengths. First, it integrates multiple analytical approaches, including pairwise meta-analysis, network meta-analysis, TSA, and PI, allowing for a more comprehensive assessment of both effect size and robustness. Second, it includes a larger and more up-to-date body of RCT evidence across diverse DHI types and outcomes. Third, unlike previous studies that focused on single interventions or relied primarily on CIs, this systematic review explicitly accounts for heterogeneity and real-world uncertainty, providing a more nuanced and clinically relevant interpretation of the findings [20-24]. These features enhance the methodological rigor, comprehensiveness, and applicability of the evidence. In line with these methodological advantages, the network meta-analysis further enabled comparisons across different DHIs. Interestingly, interventions involving electronic adherence monitoring devices did not demonstrate clear advantages for key clinical outcomes such as viral suppression. Similar observations have been reported in previous systematic reviews, suggesting that passive monitoring alone may be insufficient to produce substantial improvements in clinical outcomes [114]. Therefore, combining monitoring technologies with active behavioral interventions may represent a more effective strategy.

Nevertheless, the overall certainty of evidence ranged from low to very low for most outcomes. While DHIs may offer some benefits, the current evidence remains insufficient to support definitive conclusions. Variability in study quality, sample size, and methodology likely contributes to this uncertainty and highlights the need for more robust and well-designed trials. Accordingly, these findings should be interpreted with caution, as future research may alter the current understanding of DHI effectiveness.

### Limitations

Several limitations should be considered when interpreting the findings of this review. First, the methodological quality of the included studies varied substantially, and a considerable proportion of trials were judged to have some concerns or a high risk of bias, which may have influenced the pooled estimates. Second, substantial clinical and methodological heterogeneity existed across the included studies, including differences in intervention types, population characteristics, and treatment-era policies. These factors may influence the real-world effectiveness of DHIs. Because the reporting of intervention details and study-level variables was not fully consistent across studies, more detailed exploratory analyses were not performed. Future studies with more standardized reporting of intervention characteristics and study designs would help further investigate potential sources of heterogeneity. Third, potential small-study effects were detected in the analysis of objective adherence outcomes, and trim-and-fill adjustments suggested the possible presence of publication bias. Fourth, although the classification framework included multiple types of digital interventions, only a subset was supported by a relatively large number of RCTs. Other categories, such as mobile app interventions, were represented by fewer studies, limiting the certainty of evidence for these DHIs. Fifth, studies involving multiple digital components were grouped into a single category of multiple digital interventions, which may have introduced additional clinical heterogeneity and potentially obscured differences among specific intervention components. Sixth, this review included only RCTs. While this approach strengthens internal validity, it may limit the inclusion of real-world implementation evidence [115,116], which is particularly important for DHIs deployed in routine care settings. Finally, evidence from Asia and Europe was relatively limited.

### Conclusions

This systematic review integrates the most recent RCTs and provides a comprehensive evaluation of the effects of DHIs on both behavioral and clinical outcomes in people living with HIV, differing from previous systematic reviews that primarily focused on single intervention types or limited outcome measures. Compared with standard care, DHIs were associated with improvements in viral suppression, treatment adherence, and retention, but showed no significant effect on CD4^+^ cell counts. These findings support the potential role of DHIs as nonpharmacological interventions for people living with HIV. This systematic review provides a comprehensive evidence base that may inform the design of future research and guide the cautious implementation of DHIs in clinical practice. However, the real-world applicability of these findings remains uncertain. The wide 95% PIs, together with potential risk of bias, small-study effects, and low to very low certainty of evidence based on GRADE, suggest substantial variability in effectiveness. Therefore, these results should be interpreted with caution, and further well-designed, high-quality studies are warranted.

## Supplementary material

10.2196/81019 Multimedia Appendix 1 Supplementary search strategy, risk-of-bias assessment, trial sequential analyses, subgroup analyses, funnel plots, study characteristics, and sensitivity analyses.

10.2196/81019 Checklist 1 PRISMA checklist 2020.

10.2196/81019 Checklist 2 PRISMA 2020 for abstracts checklist.

10.2196/81019 Checklist 3 PRISMA-S checklist.

## Abbreviations

ART: antiretroviral therapy
DHI: digital health intervention
MD: mean difference
PI: prediction interval
PRISMA: Preferred Reporting Items for Systematic Reviews and Meta-Analyses
PRISMA-S: Preferred Reporting Items for Systematic Reviews and Meta-Analyses literature search extension
RCT: randomized controlled trial
RIS: required information size
RoB 2: Risk of Bias 2
RR: risk ratio
SOC: standard of care
TSA: Trial sequential analysis
