# Supplementary material for: Effectiveness of Digital Health Interventions for Improving Antiretroviral Therapy Outcomes in People With HIV: Meta-Analysis and Trial Sequential Analysis of Randomized Controlled Trials
Source: J Med Internet Res. 2026 Jul 23;28:e81019. doi: 10.2196/81019 (PMC13394862; doi:10.2196/81019)
Supplement: Multimedia Appendix 1 [file jmir-v28-e81019-s001.docx]

**Supplement**

[**Search strategy 2**](#_Toc173832379)

[**Figure S1. Risk of bias 6**](#_Toc173832379)

[**[Figure S2. TSA 7](#_Toc173832380)**](#_Toc173832379)

**[Figure S3. Subgroup analysis 1](#_Toc173832380)0**

[**Figure S4. Change of CD4+ cell level 1**](#_Toc173832381)**4**

[**Figure S5. objective adherence. 1**](#_Toc173832382)**4**

[**Figure S6. Retention 1**](#_Toc173832383)**5**

[**Figure S7. Funnel plot 1**](#_Toc173832384)**6**

[**Table S1. Supplementary Information Form 1**](#_Toc173832384)**8**

[**Table S2. Sensitivity Analysis 4**](#_Toc173832384)0

Search strategy

PubMed

1."HIV"[Mesh] OR "HIV Infections"[Mesh] OR HIV[tiab] OR "Human Immunodeficiency Virus*"[tiab] OR "AIDS Virus*"[tiab]

2. "Acquired Immunodeficiency Syndrome"[Mesh] OR AIDS[tiab] OR "Acquired Immunodeficiency Syndrome"[tiab] OR "Acquired Immune Deficiency Syndrome"[tiab]

3. #1 OR #2

4. "Mobile Applications"[Mesh] OR "Telemedicine"[Mesh] OR "Text Messaging"[Mesh] OR "Cell Phone"[Mesh] OR "Internet"[Mesh]

5. mHealth[tiab] OR "mobile health"[tiab] OR eHealth[tiab] OR "digital health"[tiab] OR telemedicine[tiab] OR telehealth[tiab] OR "mobile application*"[tiab] OR "health app*"[tiab] OR smartphone*[tiab] OR "cell phone*"[tiab] OR "text messag*"[tiab] OR SMS[tiab] OR "short message"[tiab] OR "web-based"[tiab] OR "internet-based"[tiab] OR "online intervention*"[tiab] OR "digital intervention*"[tiab]

6. "Medication Adherence"[Mesh] OR "Patient Compliance"[Mesh]

7. adherence[tiab] OR compliance[tiab] OR "medication adherence"[tiab] OR "treatment adherence"[tiab] OR "drug adherence"[tiab] OR persistence[tiab] OR "medication taking"[tiab]

8. #6 OR #7

9. #4 OR #5

10. #3 AND #9 AND #8

Cochrane Library

1. MeSH descriptor: [HIV] explode all trees
   2. MeSH descriptor: [HIV Infections] explode all trees
   3. (HIV OR "Human Immunodeficiency Virus*" OR "AIDS Virus*"):ti,ab
   4. #1 OR #2 OR #3

   5. MeSH descriptor: [Acquired Immunodeficiency Syndrome] explode all trees
   6. (AIDS OR "Acquired Immunodeficiency Syndrome" OR "Acquired Immune Deficiency Syndrome"):ti,ab
   7. #5 OR #6
   8. #4 OR #7
2. MeSH descriptor: [Mobile Applications] explode all trees
   10. MeSH descriptor: [Telemedicine] explode all trees
   11. MeSH descriptor: [Text Messaging] explode all trees
   12. MeSH descriptor: [Cell Phone] explode all trees
   13. MeSH descriptor: [Internet] explode all trees
   14. (mHealth OR "mobile health" OR eHealth OR "digital health" OR telemedicine OR telehealth OR "mobile application*" OR "health app*" OR smartphone* OR "cell phone*" OR "text messag*" OR SMS OR "short message" OR "web-based" OR "internet-based" OR "online intervention*" OR "digital intervention*"):ti,ab
   15. #9 OR #10 OR #11 OR #12 OR #13 OR #14
3. MeSH descriptor: [Medication Adherence] explode all trees
   17. MeSH descriptor: [Patient Compliance] explode all trees
   18. (adherence OR compliance OR "medication adherence" OR "treatment adherence" OR "drug adherence" OR persistence OR "medication taking"):ti,ab
   19. #16 OR #17 OR #18

20. #8 AND #15 AND #19

Embase

1. 'human immunodeficiency virus'/exp OR 'hiv infection'/exp

2. (HIV OR "Human Immunodeficiency Virus*" OR "AIDS Virus*"):ti,ab

3. #1 OR #2

4. 'acquired immune deficiency syndrome'/exp

5. (AIDS OR "Acquired Immunodeficiency Syndrome" OR "Acquired Immune Deficiency Syndrome"):ti,ab

6. #4 OR #5

1. #3 OR #6

8. 'mobile application'/exp OR 'telemedicine'/exp OR 'text messaging'/exp OR 'cell phone'/exp OR 'internet'/exp

9. (mHealth OR "mobile health" OR eHealth OR "digital health" OR telemedicine OR telehealth OR "mobile application*" OR "health app*" OR smartphone* OR "cell phone*" OR "text messag*" OR SMS OR "short message" OR "web-based" OR "internet-based" OR "online intervention*" OR "digital intervention*"):ti,ab

10. #8 OR #9

11. 'medication compliance'/exp OR 'patient compliance'/exp

12. (adherence OR compliance OR "medication adherence" OR "treatment adherence" OR "drug adherence" OR persistence OR "medication taking"):ti,ab

13. #11 OR #12

14. #7 AND #10 AND #13

1. TS=(HIV OR "Human Immunodeficiency Virus*" OR "AIDS Virus*")

2. TS=("Acquired Immunodeficiency Syndrome" OR AIDS OR "Acquired Immune Deficiency Syndrome")

3. #1 OR #2

4. TS=("mobile application*" OR "health app*" OR mHealth OR "mobile health" OR eHealth OR "digital health" OR telemedicine OR telehealth OR smartphone* OR "cell phone*" OR "text messag*" OR SMS OR "short message" OR "web-based" OR "internet-based" OR "online intervention*" OR "digital intervention*")

5. TS=(adherence OR compliance OR "medication adherence" OR "treatment adherence" OR "drug adherence" OR persistence OR "medication taking")

6. #3 AND #4 AND #5

**Figure S1. Risk of bias**


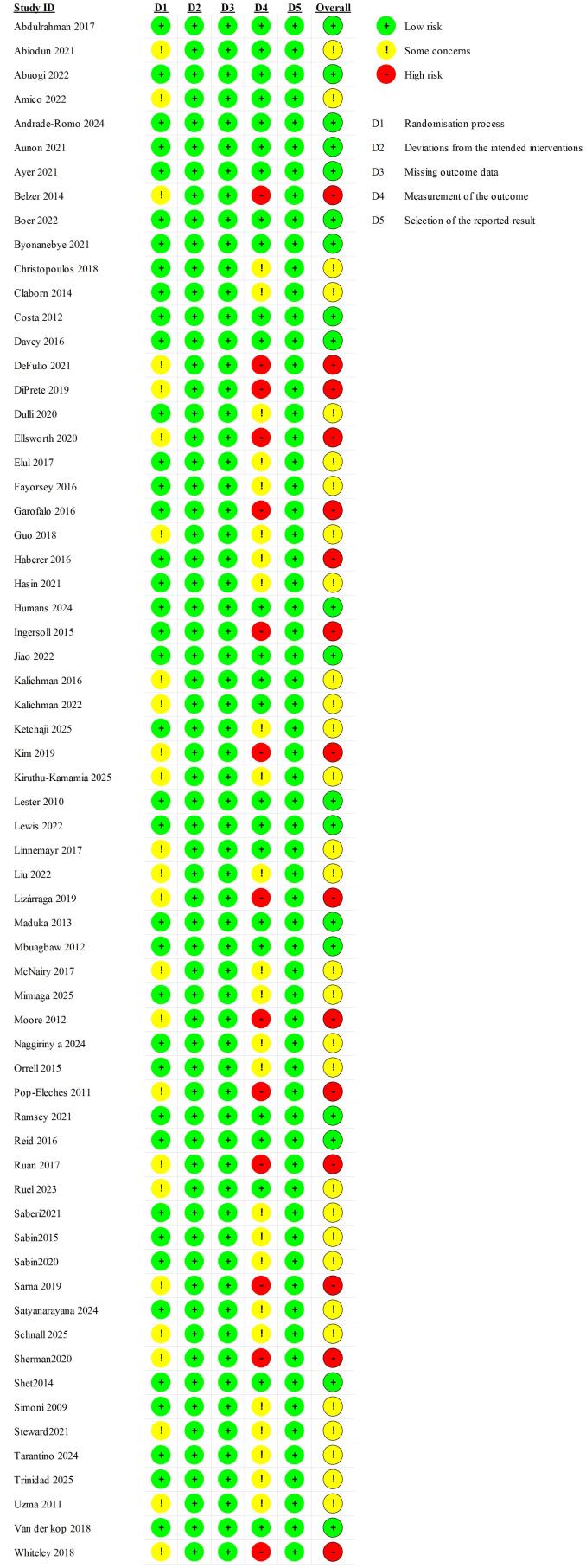


**Figure S2. TSA**

**Figure S2.1. Virus suppression**


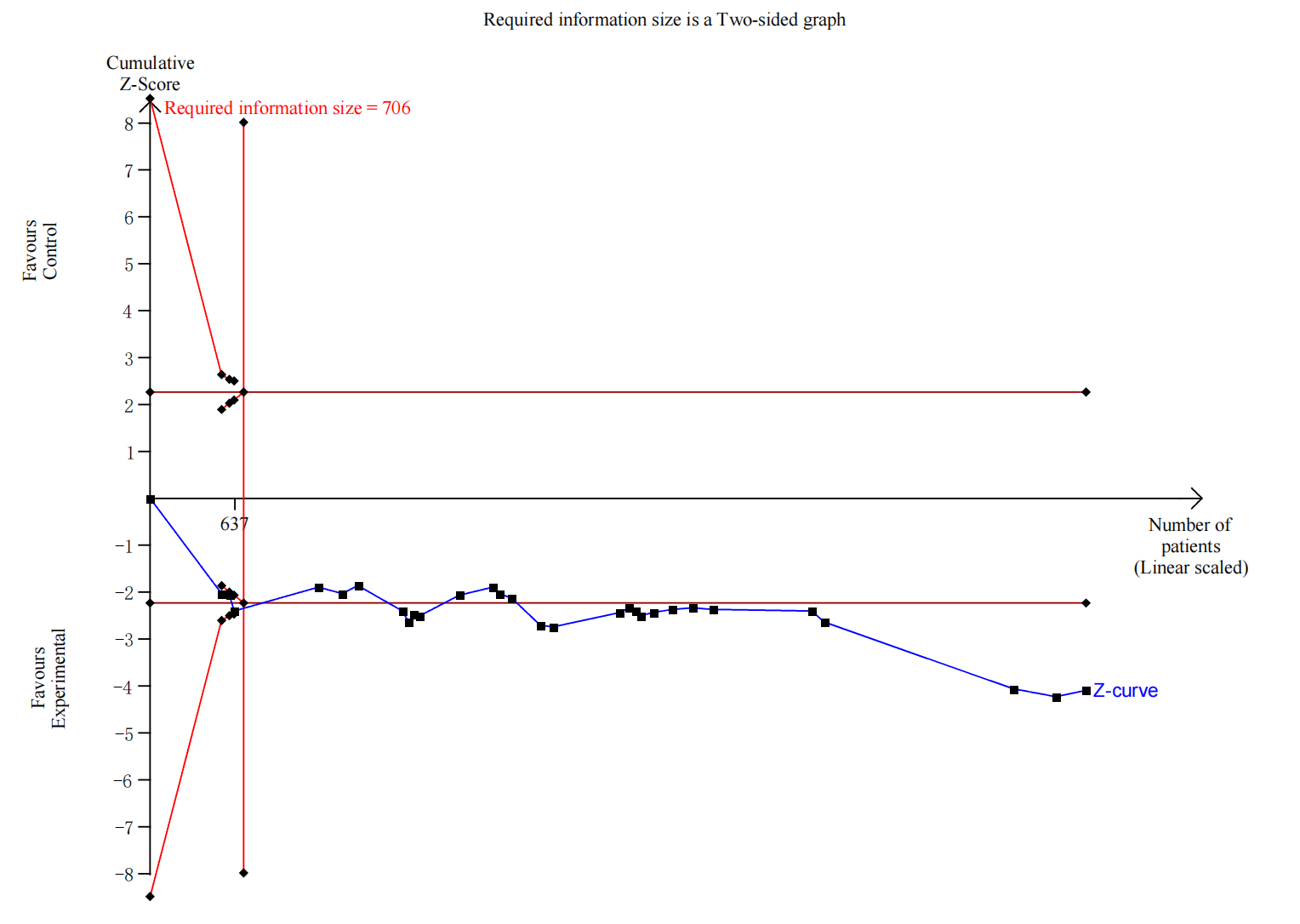


**Figure S2.2. CD4+ cell level**


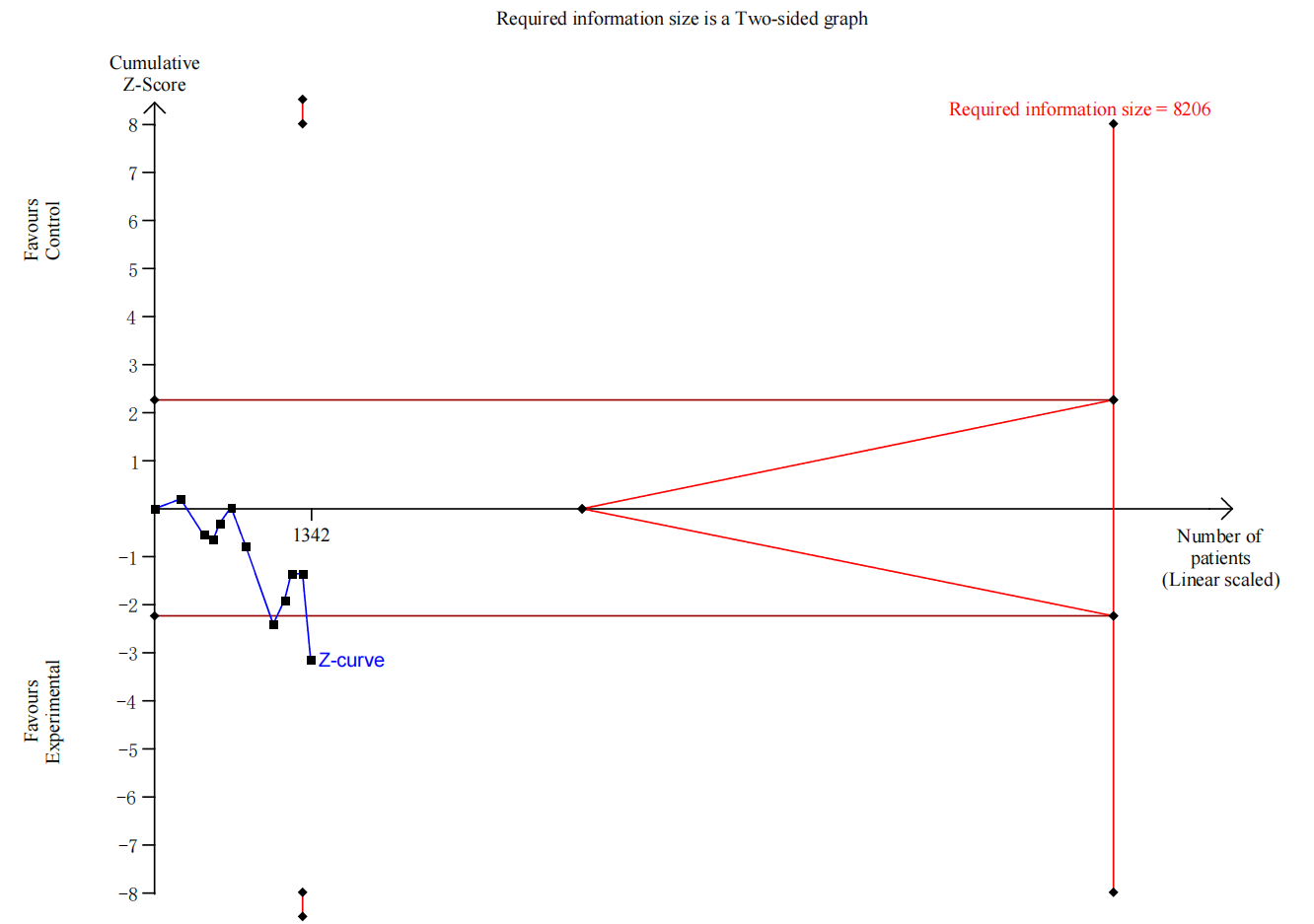


**Figure S2.3. Subjective adherence**


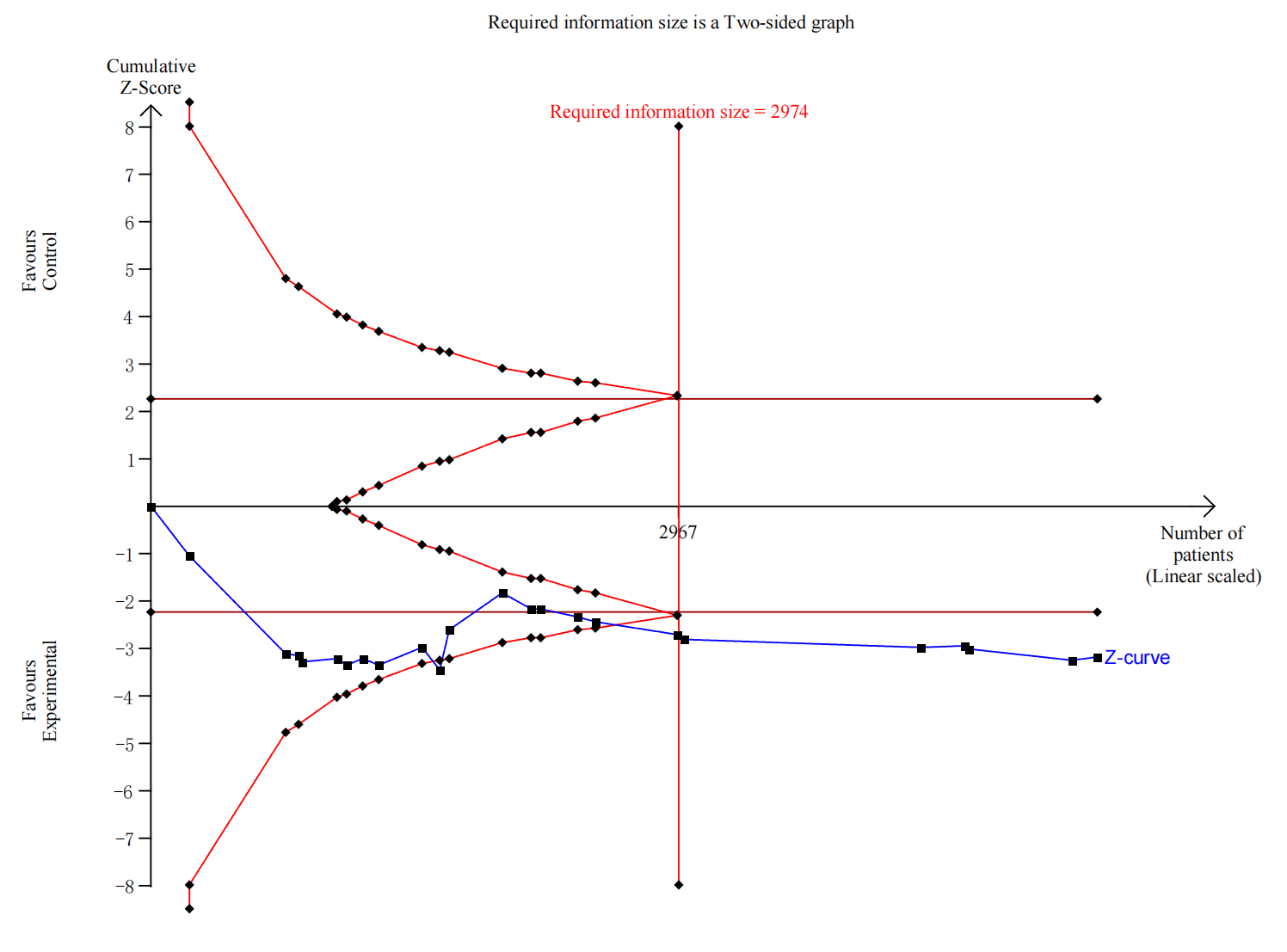


**Figure S2.4. Objective adherence**


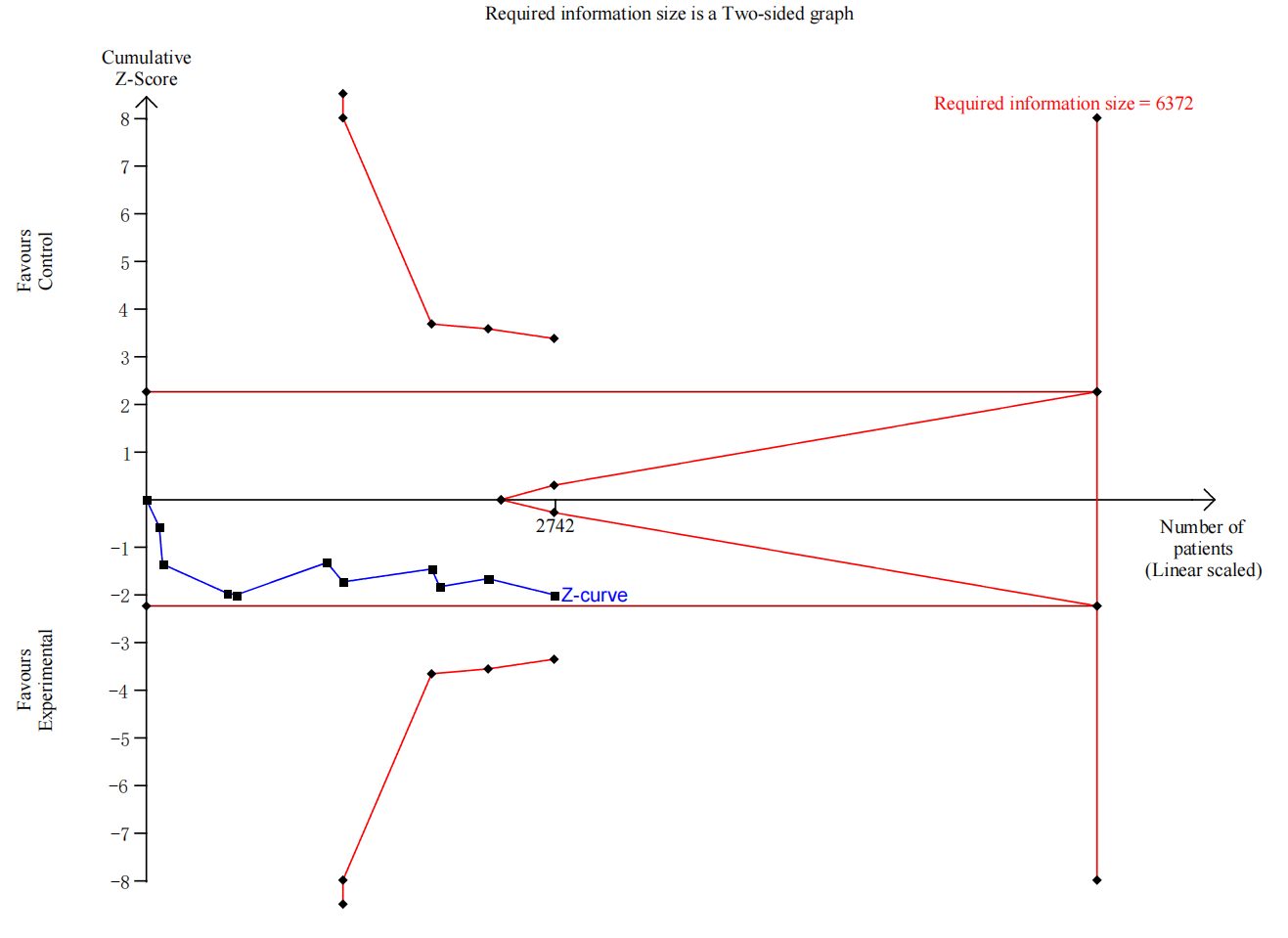


**Figure S2.5. Retention**


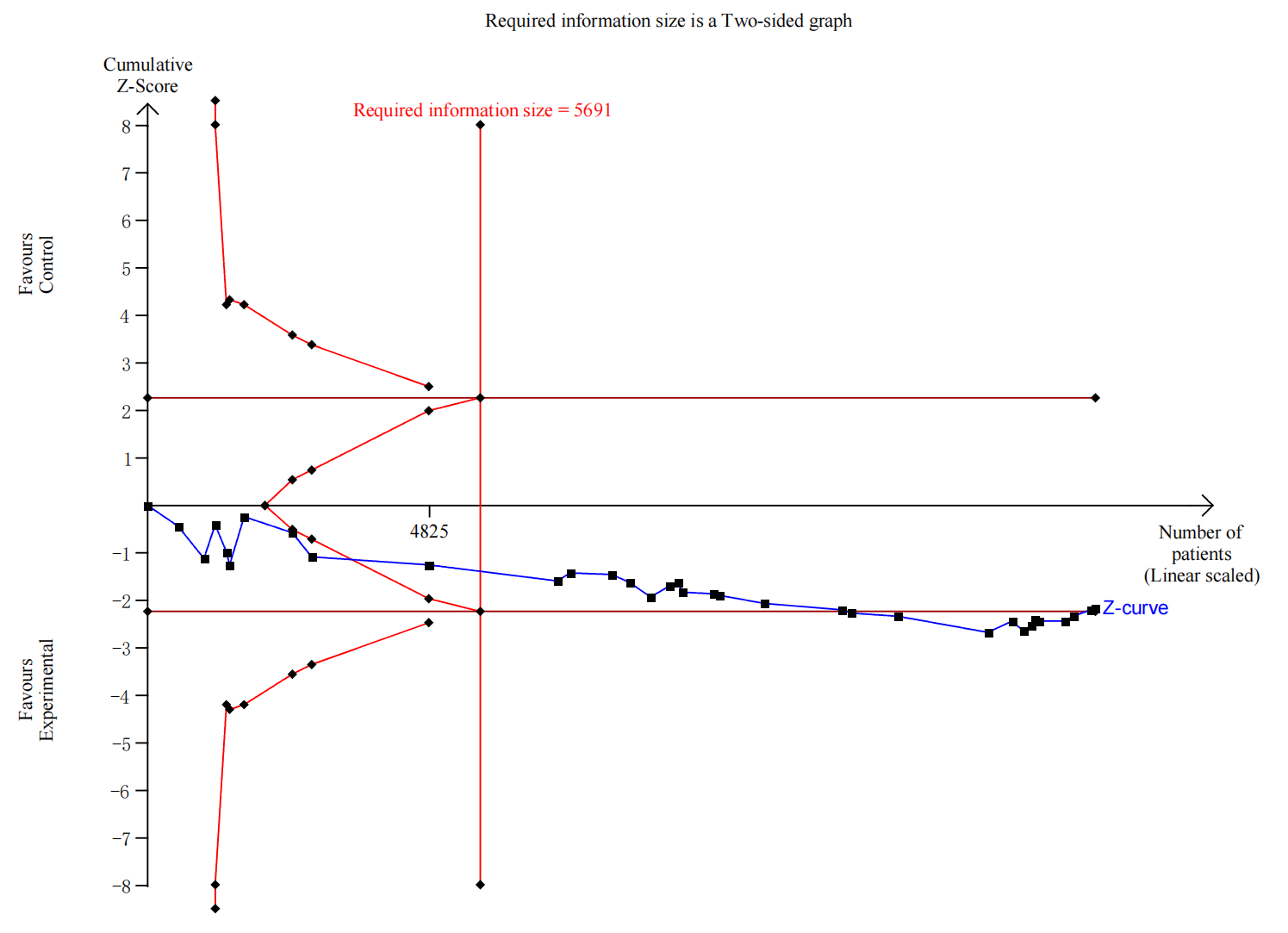


**Figure S3. Subgroup analysis**

**Figure S3.1. Virus suppression**


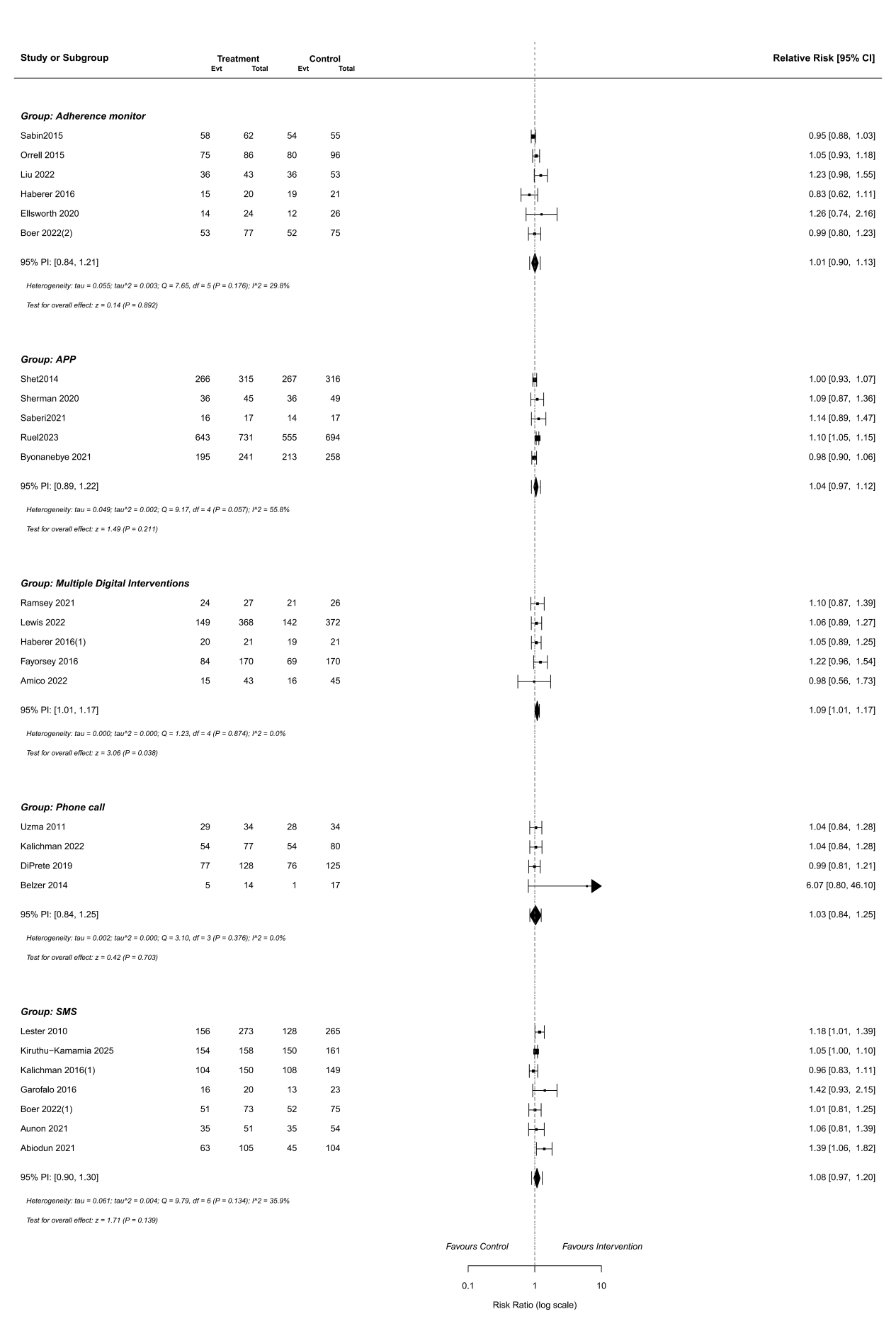


**Figure S3.2. subjective adherence**


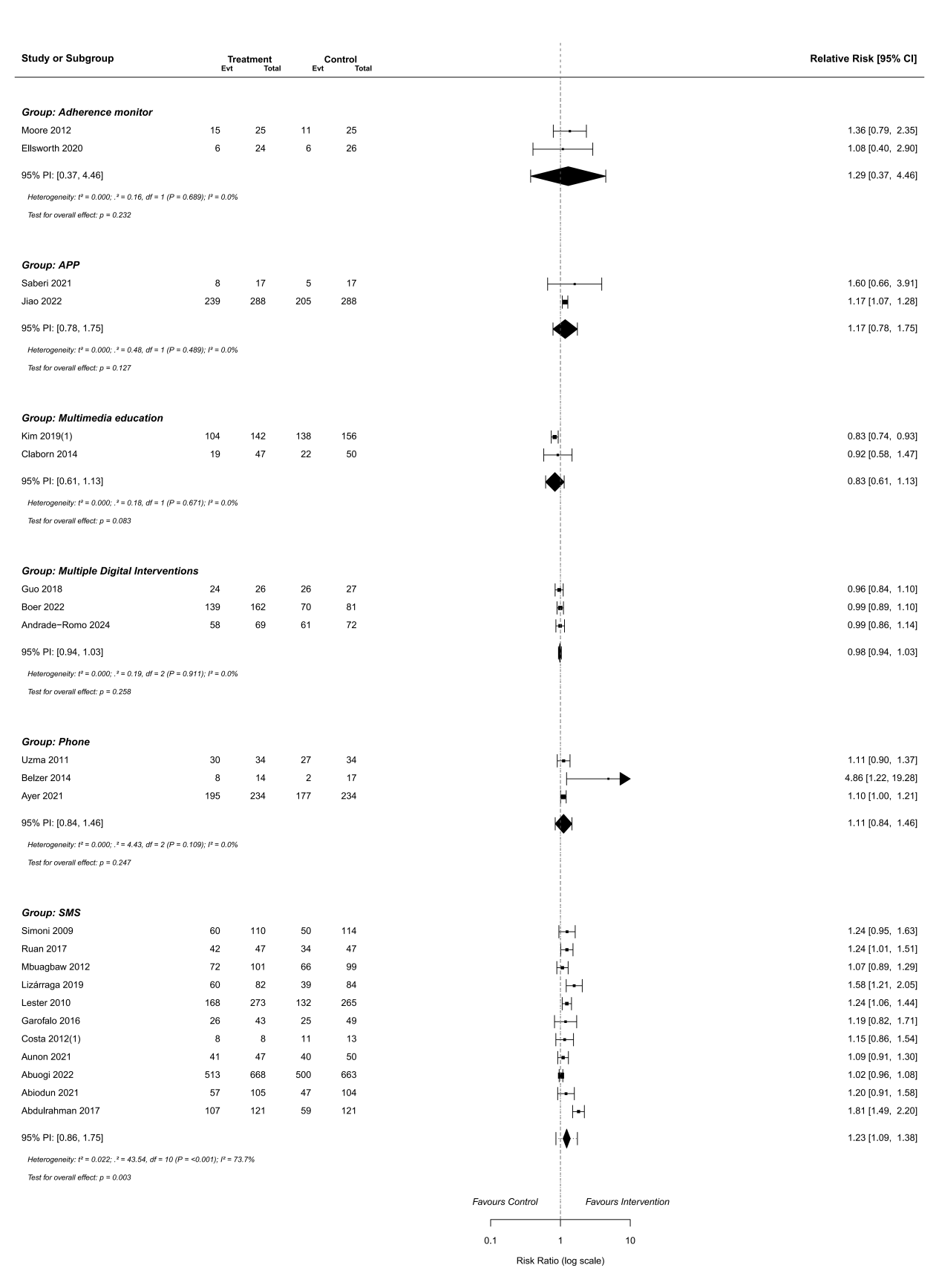


**Figure S3.3. Objective adherence**


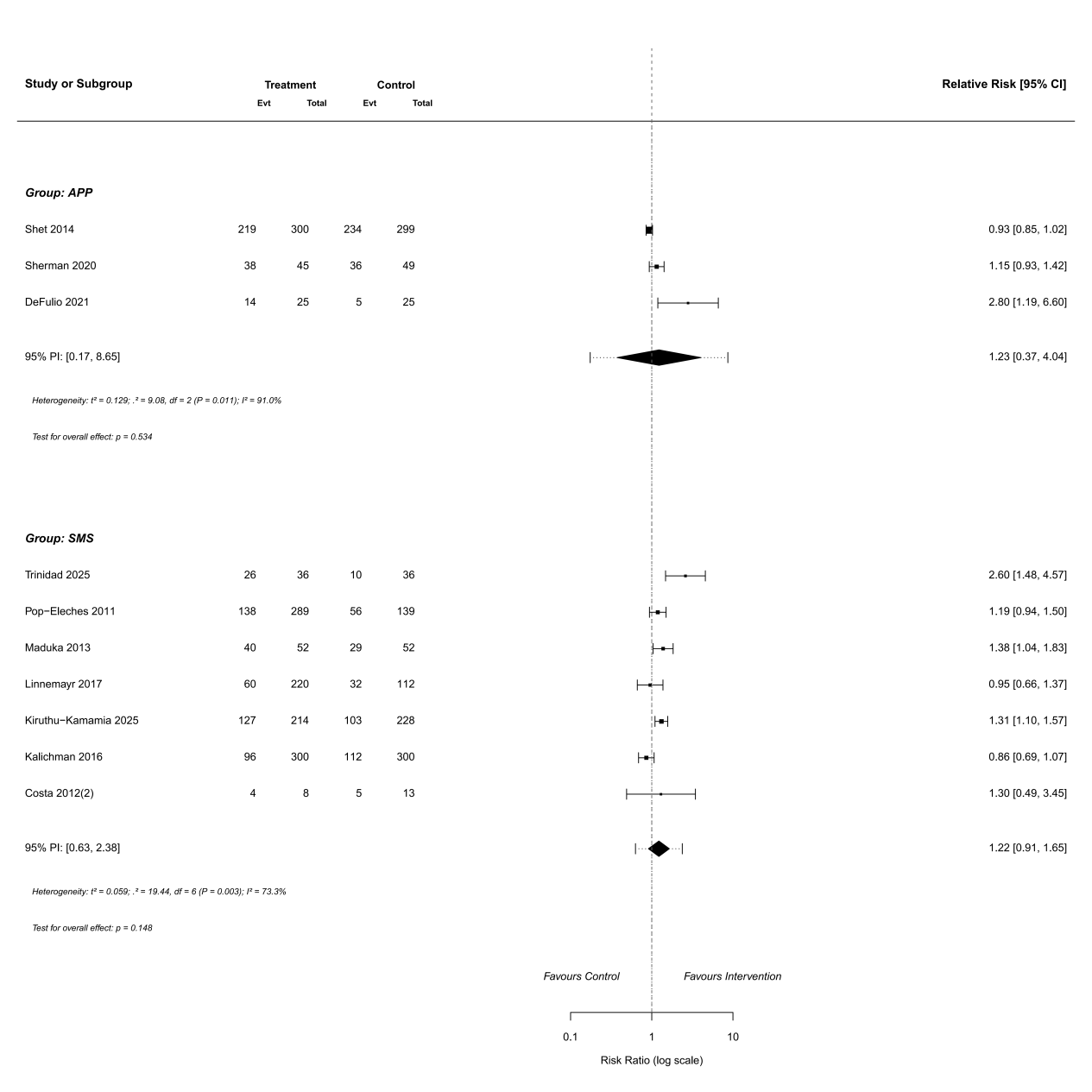


**Figure S3.4. Retention**


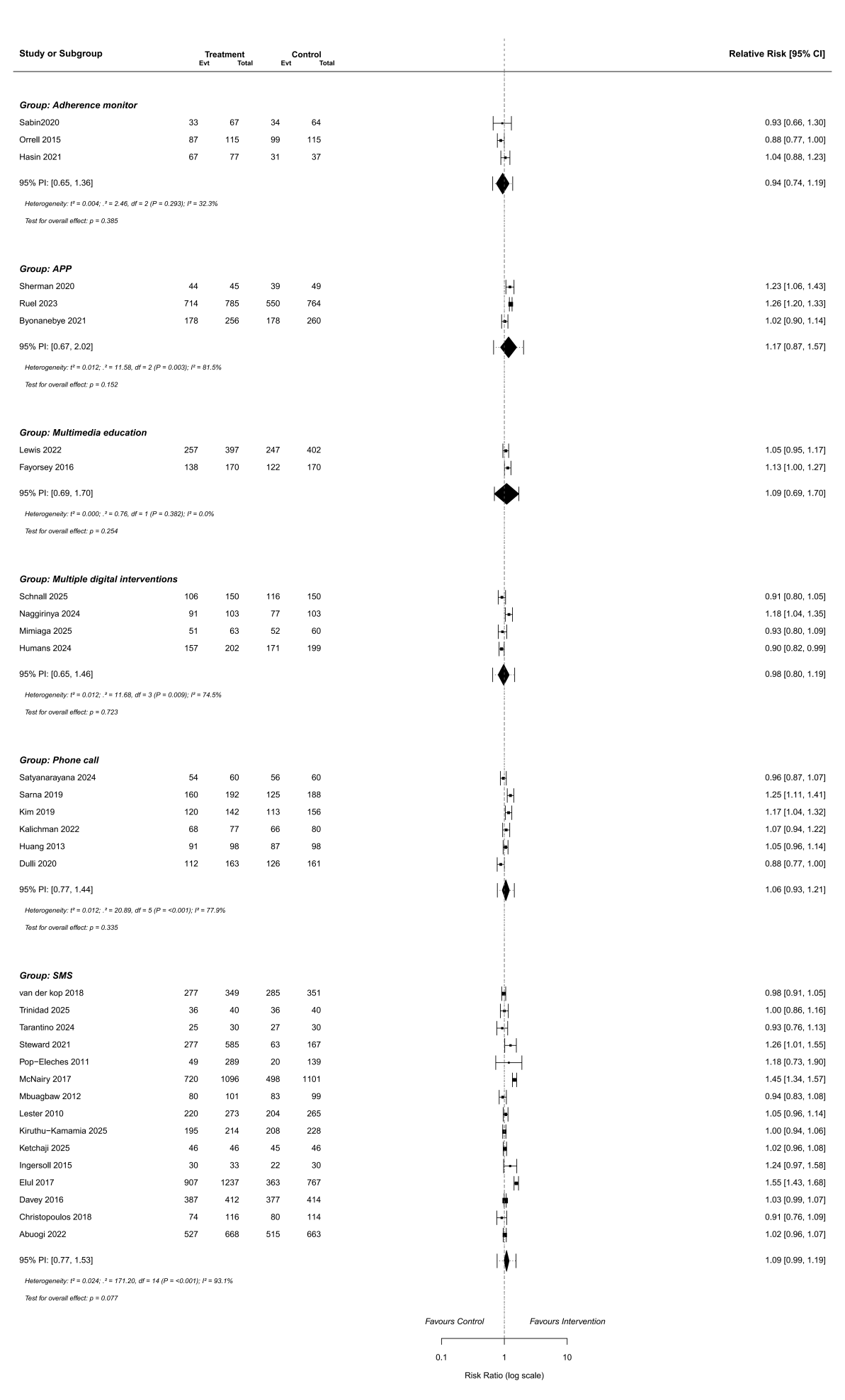


**Figure S4. CD4+ cell**


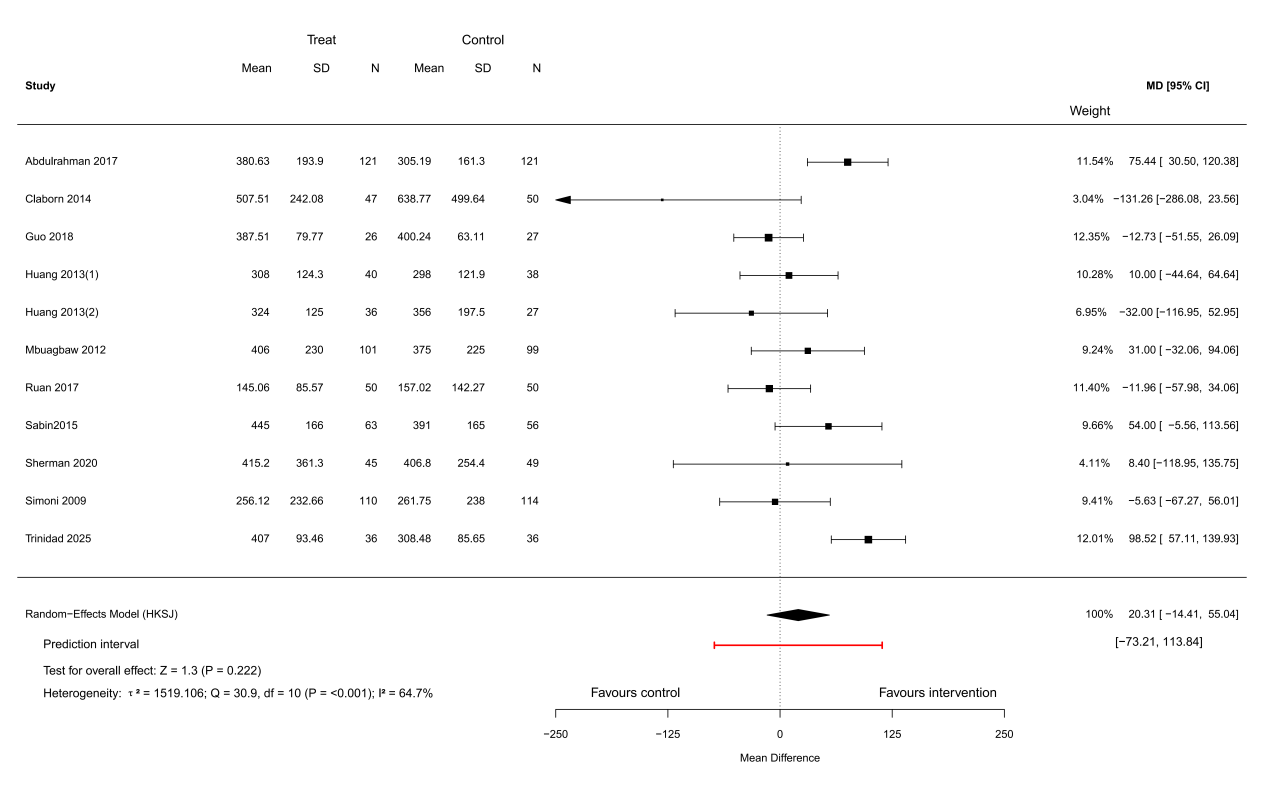


**Figure S5. Objective adherence**


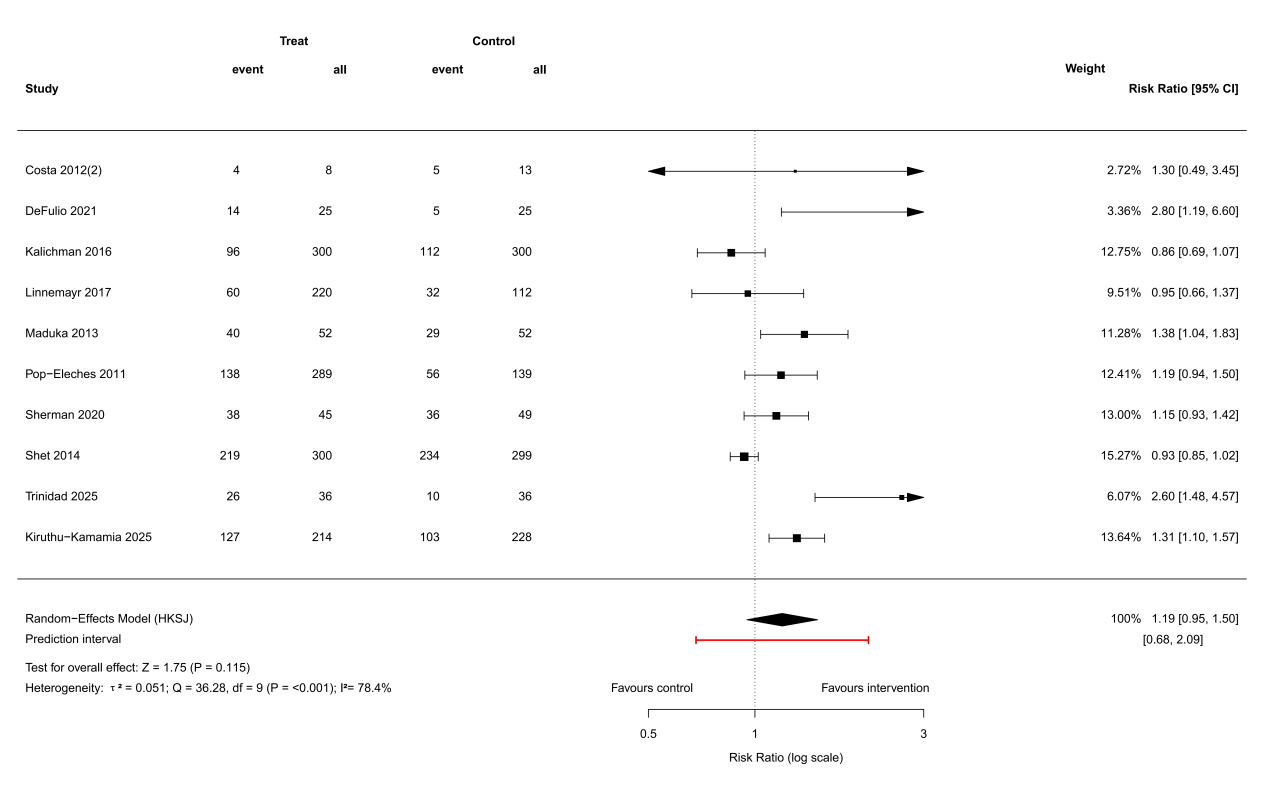


**Figure S6. Retention**


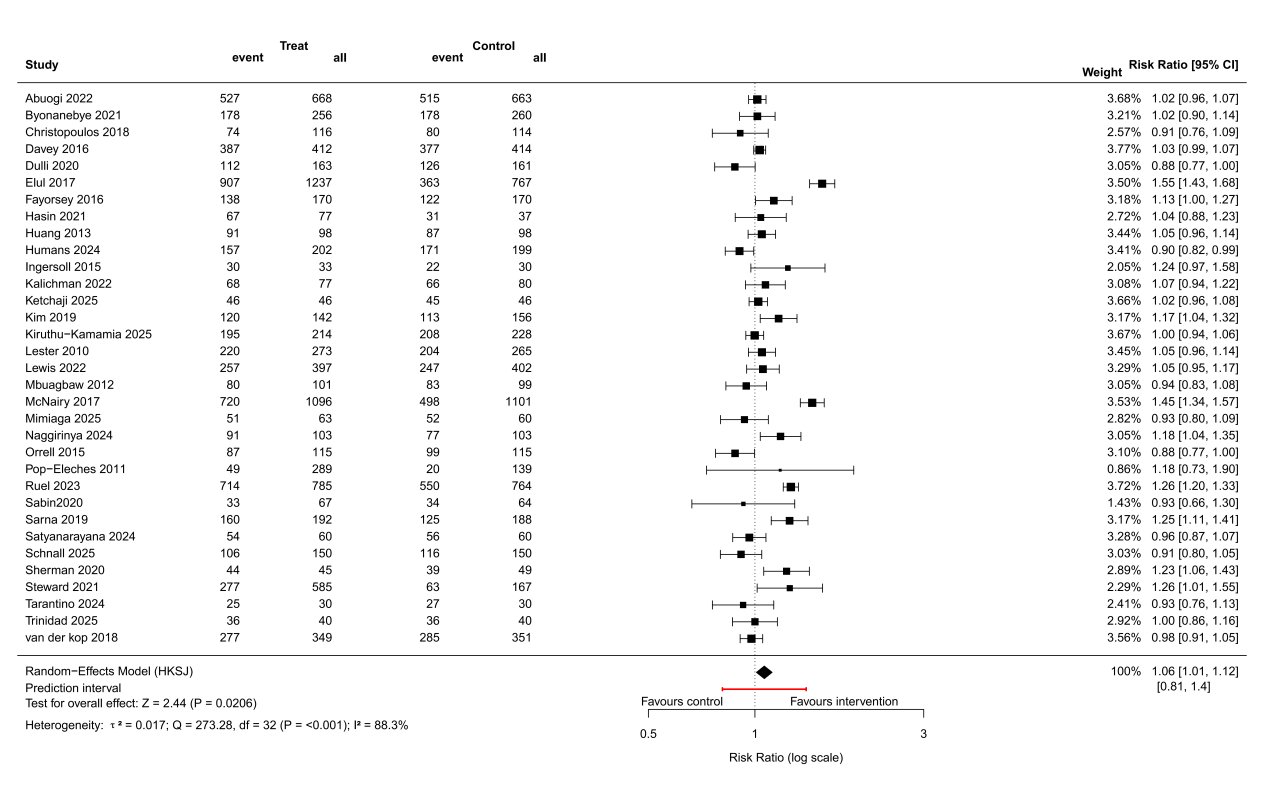


**Figure S7. Funnel plot**

**Figure S7.1. Virus suppression**

Prob > |z| = 0.5566

**Figure S7.2. CD4+ cell level**

Prob > |z| = 0.0007

| Studies | Hedges's g | [95% conf. | interval] |
| --- | --- | --- | --- |
|  |  |  |  |
| Observed | -0.613 | -0.812 | -0.415 |
| Observed=+Imputed | -0.482 | -0.702 | -0.263 |

**Figure S7.3. Subjective adherence**

 Prob > |z| = 0.4606

**Figure S7.4. Objective adherence**

Prob > |z| = 0.0040

| Studies | Hedges's g | [95% conf. | interval] |
| --- | --- | --- | --- |
|  |  |  |  |
| Observed | 0.46 | 0.023 | 0.897 |
| Observed=+Imputed | 0.081 | -0.428 | 0.59 |

**Figure S7.5. Retention**

Prob > |z| = 0.8295

**Table S1. Supplementary Information Form**

| **Study** | **Study design; duration**  **(mo)** | **Intervention** | **Outcomes** | | | **Conclusion** |
| --- | --- | --- | --- | --- | --- | --- |
|  |  |  | **Virus suppression** | **Adherence** | **Retention** |  |
| **SMS** |  |  |  |  |  |  |
| Abdulrahman 2017 | RCT;6 | Weekly text reminders for medication adherence, appointment reminders 2 days before follow-up, and at least three personal counseling sessions. | Viral suppression: Intervention group 99.1%, Control group 89.3%, p = 0.028; Log VL: Intervention 1.41, Control 1.76. | Control group 54.6% vs. Intervention group 92.2% with good adherence (>95%), p = 0.001. | NA | Effective |
| Abiodun 2021 | RCT;12 | Two days before follow-up dates, receive a reminder text for follow-up appointments. Daily ART adherence reminder texts, with responses required. | Viral suppression: Control 44.23% vs. Intervention 60%, p = 0.022; Log VL: 2.63 [1.55] vs. 1.97 [1.30], p = 0.001. | Control group 45.19% vs. Intervention group 54.29%, p = 0.189. | NA | Effective |
| Abuogi 2022 | RCT;12 | Community mentor mothers + weekly messaging focused on medication and clinic adherence. | 12-month viral suppression: 5.4% not suppressed (p < 0.001). | Control group 97.1% vs. Intervention group 97.6%. | Control group 77.7% vs. Intervention group 78.9%. | Ineffective for adherence and retention |
| Aunon 2021 | RCT;12 | TextIt platform automatically sends motivational and behavioral skills-related text messages. | Intervention group 69% vs. Control group 63%, p = 0.52. | First month: Intervention group 93.0% vs. Control 71.2%, p = 0.01. | NA | Effective for short-term adherence |
| Boer 2022(1) | RCT;10 | SMS group receives random reminders on 3 days per week, with a requirement to reply. Real-time medication monitoring group gets text reminders if pillbox is not opened on time. | SMS 70%, Medication 69%, Control 69%, p = 0.99. | SMS 90%, Pillbox 85%, Control 86%, p = 0.64. | NA | Ineffective |
| Christopoulos 2018 | RCT;6 | The C4C intervention messages were delivered via an automated SMS platform, which also provided appointment reminders and research follow-up messages/calls. | Intervention group 44% vs. Control group 39.5%. | NA | Intervention group 63.4% vs. Control group 70.6%. | Ineffective for viral suppression |
| Costa 2012 | RCT;6 | HIVAS network platform automatically sends medication reminders. Messages sent on Saturdays, Sundays, and alternate weekdays. | NA | Self-reported adherence: Intervention group 100.00% vs. Control 84.62%, p = 0.24. Pill count adherence: Intervention 50.00% vs. Control 38.46%, p = 0.24. | NA | Effective |
| Davey 2016 | RCT;12 | SMSaúde platform sends general messages, appointment reminders, medication reminders, and educational messages. | NA | NA | Intervention group 93.8% vs. Control group 91.0%, p = 0.14. | Ineffective |
| Elul 2017 | RCT;12 | Health information and appointment reminders received via SMS. Weekly for the first month, then monthly. Appointment reminders sent 3-7 days before clinic visits. | NA | NA | CIS: 74%, CIS+: 73%, Control 47%. | Effective |
| Garofalo 2016 | RCT;12 | Remedy Health Media provides daily text reminders to the intervention group for 6 months. | Log VL: Intervention group mean (SD) 2.2 (1.4) vs. Control group mean (SD) 2.2 (1.0); Suppression: Intervention 80%, Control 56.5%. | Intervention 60.5% vs. Control 51.0%, significant difference. | NA | Effective for adherence |
| Ingersoll 2015 | RCT;12 | Bidirectional text messaging system, sending daily queries about medication dosage. | NA | Intervention 85% vs. Control 71%, p = 0.04. | Intervention group 91% vs. Control group 72%, p = 0.12. | Effective |
| Kalichman 2016(1) | RCT;12 | Face-to-face office visits followed by four biweekly counseling sessions via phone. Up to two daily medication time reminders. Contact-matched non-contaminated health improvement interventions. | Adherence Counseling 79% vs. Adherence Counseling + Reminders 79% vs. Contact 79% vs. Matched Control 73% vs. Contact Matched + Reminders 76%; Adherence counseling significant, p < 0.05. SMS effect and counseling × SMS interaction not significant. | Adherence Counseling 43%, Adherence Counseling + Reminders 43%, Matched Control 42%, Contact Matched + Reminders 34%, significant adherence counseling, p < 0.05. SMS effects, counseling × SMS interaction not significant. | NA | Effective |
| Lester 2010 | RCT;1 | Weekly texts sent to intervention group patients, with instructions to respond within 48 hours. Clinicians call patients with issues or those who do not respond within 2 days. | Intervention 57% vs. Control 48%, p = 0.04. | Intervention 62% vs. Control 50%, p = 0.006. | Intervention 81% vs. Control 77%, p = 0.31. | Effective |
| Linnemayr 2017 | RCT;5 | Weekly Sunday texts requesting replies. Non-responders receive a follow-up message. Study coordinators help identify reasons for participants' discomfort. | NA | Unidirectional group 28%, p = 0.85; Bidirectional group 26%, p = 0.69; Control group 29%. | NA | Ineffective |
| Lizárraga 2019 | RCT;12 | Two appointment reminders sent: one day before and three days before each appointment. Two weekly messages for 24 weeks. The first message encourages adherence, and the second alternates between motivational and general health information. | NA | Intervention 73.2% vs. Control 46.4%, p < 0.001. | NA | Effective |
| Maduka 2013 | RCT;1 | Weekly two text messages containing adherence-related information and HAART medication reminders. Monthly adherence counseling. | Post-intervention median CD4+ count: Control 361.5 cells/ml vs. Intervention 578.0 cells/ml, p = 0.007. | Intervention 76.9% vs. Control 55.8%, p = 0.022. | NA | Effective |
| Mbuagbaw 2012 | RCT;6 | Weekly motivational text messages with reminder components. | Post-intervention CD4+ count mean (SD): Intervention 406 (230) vs. Control 375 (225), p = 0.337. | Intervention 71.3% vs. Control 66.7%, p = 0.542. | Intervention 79.2% vs. Control 83.8%, p = 0.399. | Ineffective |
| McNairy 2017 | RCT;12 | Appointment reminders via SMS (or voice) 3 days before scheduled appointments. All patients receive SMS (or voice) reminders within 7 days after missed appointments. | HIV-1 RNA < 1,000 copies/ml: Intervention 88% vs. Control 90%, p = 0.55. | NA | Intervention 66% vs. Control 45%, p = 0.002. | Effective for retention |
| Pop-Eleches 2011 | RCT;3 | Daily unidirectional reminder SMS. | NA | Intervention 47% vs. Control 46%, not significant, p = 0.19. | Intervention 83% vs. Control 85.6%, not significant, p = 0.48. | Effective in the weekly text message subgroup |
| Ruan 2017 | RCT;6 | Unidirectional SMS to increase knowledge, improve medication adherence, provide emotional support, and address issues. For the first 3 months, 5 pre-loaded texts per week; last 3 months, 3 texts per week. Daily medication reminders. | CD4+ count mean (SD): Intervention 145.06±85.57 vs. Control 157.02±142.27, p = 0.623. | Control group 72.3% vs. Intervention group 89.3%, p = 0.027. | NA | Effective |
| Simoni 2009 | RCT;6 | A set of bidirectional texts; a set with peers plus bidirectional texts including medication reminders, education, entertainment, and adherence assessment. | CD4+ count mean (SD): Intervention 256.1±232.7 vs. Control 261.8±238. | Intervention 54.5% vs. Standard treatment 43.9%. | NA | Effective |
| Steward2021(1) | RCT;3 | Appointment reminders before the visit, and follow-up reminders every two weeks after a missed appointment until the participant returns to care or after three months. Biweekly brief behavioral information and SMS check-in messages. Navigator meets patients monthly and handles appointment scheduling via phone or SMS. | NA | NA | Intervention 47.27% vs. Standard treatment 37.72%. | Effective |
| Trinidad 2025 | RCT;6 | Participants received two messages per week (Monday and Thursday mornings) during the first three months, then one message per week during the last three months. During the 6-month intervention period, they also received reminder messages 24 hours before scheduled appointments or prescription refills. | NA | Control (72%) vs. Intervention (28%), p = p< 0.001. | Intervention 90% vs. Standard treatment 90%. | Effective |
| Tarantino 2024 | RCT;12 | [A text messaging app was developed using the web-based CommCare platform. Messages were primarily two-way, automated, thematic, and personalized, and were categorized as adherence, skills, motivational, or story-based messages.](https://link.springer.com/article/10.1007/s10461-024-04560-3#ref-CR23) | NA | NA | Intervention 83.3% vs. Controlt 90%. | Effective |
| Ketchaji 2025 | RCT;6 | Messages were sent daily via the local telecom network, 30 minutes before ART dosing. | NA | Control 85%, SMS 27% | Intervention 100% vs. Controlt 97.8%. | Effective |
| Kiruthu-Kamamia 2025 | RCT;6 | Participants in the intervention group received automated weekly motivational messages and personalized SMS appointment reminders 3 days and 1 day before scheduled visits, as well as follow-up reminders at 2, 5, and 11 days after a missed appointment (if applicable). In addition, the intervention supported open two-way SMS communication between clients and retention officers. | Intervention 97.5% vs. Control 93.2%. | Control 46%, SMS 59.6% | Intervention 91.1% vs. Controlt 91.2%.p = 0.07 | Effective |
| van der kop 2018 | RCT;9 | Weekly Monday texts requesting responses. Non-responders are called and reasons for non-response are documented. | NA | NA | Intervention 79% vs. Control 81%, p = 0.54. | Ineffective |
| **Adherence monitor** |  |  |  |  |  |  |
| Boer 2022(2) | RCT;9 | SMS group receives random reminders on 3 days per week, with a requirement to reply. Real-time medication monitoring group receives SMS reminders if pillbox is not opened on time. | SMS 70%, Medication 69%, Control 69%, p = 0.99. | SMS 90%, Pillbox 85%, Control 86%, p = 0.64. | NA | Ineffective |
| Ellsworth 2020 | RCT;12 | Smart pill bottles report non-adherent participants and send calls or SMS reminders. | Intervention 58% vs. Control 46%, p = 0.563. | Intervention 25% vs. Control 23%, p = 1.00. | NA | Ineffective |
| Haberer 2016(2) | RCT;3 | Real-time adherence monitor: Scheduled SMS reminders daily for 1 month, then weekly for 2 months. For the next 6 months, SMS reminders are sent only if no monitor signal is received within 2 hours of the expected medication time. Triggered SMS: Only sent if no monitor signal is received within 2 hours of the expected medication time. | Control 90.5%, Planned SMS group 95.2%, Triggered SMS group 75%, p = 0.14. | Control 79%, Planned SMS group 91% (p = 0.02), Triggered SMS group 79% (p = 0.90). | NA | Effective for adherence |
| Hasin 2021 | RCT;3 | Clinical guidelines and HealthCall self-monitoring and personalized feedback. | NA | NA | Control 83.8%, CG and HealthCall 94.7%, MI and HealthCall 79.5%. | Effective |
| Liu 2022 | RCT;3 | Pre-medication text reminders and personalized pre- and post-medication reminders for missed doses within 1 hour of scheduled medication time. | Log VL: Intervention group mean (SD) 1.957 (0.816) vs. Control group 2.230 (1.117), p = 0.174; Suppression rate: Intervention 83.7% vs. Control 67.9%, p = 0.123. | Week 24 adherence: Intervention mean (SD) 0.948 (0.088), Control mean (SD) 0.873 (0.224), p = 0.038. | NA | Effective for adherence |
| Moore 2012 | RCT;12 | Psychoeducational plus daily SMS, medication reminders, and emotional assessments. | NA | Control 45.5% non-adherence vs. iTAB 58.3% non-adherence, p = 0.38. | NA | Ineffective for adherence |
| Orrell 2015 | RCT;12 | SMS sent if device is not opened within 30 minutes of scheduled medication time. | Intervention 87.2% vs. Control 83.3%, p = 0.393. | Median adherence 82.1% (IQR, 56.6%-94.6%) vs. Control 80.4% (IQR, 52.8%-93.8%), no significant difference. | Intervention 75.7% vs. Control 86.1%, not significant. | Ineffective |
| Sabin 2015 | RCT;12 | SMS reminders sent if pillbox is not opened on time. | CD4 cell count: Intervention 389 vs. Control 363 cells/μL, p = 0.408. | Control 82.5% vs. Intervention 51.8%, p < 0.001. | Control 53.1% vs. Intervention 49.3%, p = 0.66. | Effective for adherence |
| Sabin 2020 | RCT;1 | SMS reminders sent if pillbox is not opened on time. | NA | NA | NA | Ineffective |
| Byonanebye 2021 | RCT;12 | Call for Life Uganda sends daily adherence IVR voice reminders (or SMS text messages) before medication times, along with appointment reminders and weekly voice calls providing educational health tips. | Viral suppression: Intervention 80.9% vs. Control 82.6% (213/258), p = 0.94. | 12-month adherence: Intervention 69.5%, Control 68.5%, p = 0.79. | NA | Ineffective |
| **App** |  |  |  |  |  |  |
| DeFulio 2021 | RCT;12 | SteadyRx mobile app used for recording and submitting video selfies. | NA | Intervention 56% vs. Control 20%, p = 0.037. | NA | Effective |
| Jiao 2022 | RCT;12 | SMS reminders sent biweekly for ART medication information, monthly for HIV clinical information, and biweekly for peer education stories. Daily medication reminders. Biweekly ART medication messages via WeChat, monthly HIV clinical messages via WeChat, and biweekly peer education videos on WeChat. QQ group created to encourage sharing ART experiences and health information. Admin shares short texts about HIV research and ART tips weekly. | Viral suppression: Intervention 94.5% vs. Control 93.0%; Detail CD4 T cell count (MD = -1.55, 95% CI -17.51 to 14.42) no significant difference. | Best ART adherence rate: Intervention 82.9% vs. Control 71.1%. | Intervention 91% vs. Control 72%, p = 0.001. | Effective for adherence |
| Saberi 2021 | RCT;6 | Online counseling providing health information, behavioral motivation, and behavioral skills. | Intervention 94.1% vs. Control 82.4%. | Control 47.1% vs. Intervention 29.4%. | NA | Ineffective for adherence and viral suppression |
| Sherman 2020 | RCT;4 | Daily unidirectional SMS reminders. | Viral suppression: Intervention 86.7% vs. Control 73.5%, p = 0.112; CD4 cell counts, mean ± SD: Intervention 415.2 ± 361.3 vs. Control 406.8 ± 254.4, p = 0.902. | Intervention 84.4% vs. Control 73.5%, p = 0.194. | NA | Effective for retention |
| Shet 2014 | RCT;6 | Weekly voice calls. | Intervention 84.4% vs. Standard treatment 84.5%, p = 0.95. | Intervention 73% vs. Standard treatment 78.3%, p = 0.13. | NA | Ineffective |
| Ayer 2021 | RCT;12 | Nurse-led phone voice call reminders monthly, sent two days before scheduled ARV pill pickup. Includes treatment-related information, counseling, and behavioral motivation. | NA | Control 84% vs. Intervention 91%, p < 0.001. | NA | Effective for adherence |
| Ruel 2023 | RCT;3 | Electronic collaborative discussions done via WhatsApp. | Intervention 88% vs. Control 80%, p = 0.0019. | NA | Intervention 97.8% vs. Control 79.6%, p = 0.006. | Effective |
| Belzer 2014 | RCT;1 | Daily or twice-daily calls with adherence facilitators, including medication reviews, problem-solving support, and arranging referrals. | Intervention (35.71) vs. Control (5.88), p = 0.043; Log10 HIV VL (copies/ml): Intervention (3.23, SD 1.40) vs. Control (4.23, SD 1.06), p = 0.043. | Control 11.76% vs. Intervention 57.14%, p = 0.046. | NA | Effective |
| **Phone call** |  |  |  |  |  |  |
| DiPrete 2019 | RCT;12 | Two motivational interviews with cognitive mapping and accompanying videos, followed by six additional treatments via phone over 12 weeks. Coordinators schedule community clinic appointments within five days of discharge and send SMS reminders before each ART dose over the first 12 weeks. | Intervention 60% vs. Control 61% in viral suppression at 24 weeks post-release, no difference between groups. | Intervention 80.3% vs. Control 81.0%, no significant difference. | Intervention 88% vs. Control 82%, no significant difference. | Ineffective |
| Huang 2013 | RCT;12 | Reminder calls every two weeks. Semi-structured dialogues to address reasons for visits, difficulties, treatment symptoms, adherence, and medication concerns. | CD4+ count in Treatment-naive patients: Intervention 111 (97.3) vs. Control 91.9 (87.8), p = 0.35; Treatment-experienced patients: Intervention 32.1 (79.0) vs. Control 12.3 (80.3), p = 0.34. | Treatment-naive patients 99.7% vs. Control 96.5%, p = 0.09; Treatment-experienced patients 99.6% vs. Control 99.5%, p = 0.37. | Intervention 83.3% vs. Control 66.5%, p < 0.001. | Ineffective |
| Kalichman 2022 | RCT;12 | Biweekly phone counseling sessions. | Intervention 74% vs. Control 72%, no difference. | NA | NA | Ineffective |
| Sarna 2019 | RCT;6 | Personalized consultations with 2 calls in the first week, then 1 call per week for up to 26 calls, followed by 2 calls in the first week postpartum and up to 16 more calls. | NA | NA | NA | Effective |
| Uzma 2011 | RCT;6 | Weekly phone reminders. | Intervention 84.2% vs. Standard treatment 82.6%, p = 0.012. | Self-reported adherence: Intervention 88.2% vs. Standard treatment 79.4%, p < 0.001. | Intervention 75.7% vs. Control 83.4%, no significant difference. | Effective |
| Claborn 2014 | RCT;24 | Combination of video and a test to assess understanding of information on HIV and medication adherence, motivational interviewing, cognitive-behavioral techniques, and improving medication adherence and clinical outcomes for HIV-positive individuals. | CD4+ count: Intervention 507.51 (242.08) vs. Control 638.77 (499.64). | One month adherence: Intervention 40.4% vs. Control 44%. | Intervention 120 vs. Control 113, p = 0.69. | Effective for adherence |
| Dulli 2020 | RCT;4 | Counselors provide information or basic counseling on ART/HIV care via Facebook groups and refer to healthcare services as needed. | NA | NA | Intervention 65% vs. Control 61%, p = 0.342. | Ineffective |
| Satyanarayana 2024 | [RCT;24](http://clinicaltrials.gov/show/NCT02319330) | Nurse-initiated mobile phone calls were conducted at participant-preferred times over 16 weeks. Calls occurred at baseline, at least twice weekly during Weeks 1–4, weekly during Weeks 5–10, and in Weeks 14 and 16, with additional calls made as needed. Nurses provided key health information and emotional support based on participant priorities. | NA | NA | Intervention 90% vs. Controlt 93.3%. | Effective |
| Kim 2019 | RCT;9 | View Mavira HIV promotional video during visits, including an 8-minute recorded Q&A on (1) starting lifelong ART while feeling healthy, (2) managing ART side effects, (3) partner disclosure. | NA | Intervention 95% vs. Control 95%, p = 0.31. | NA | Ineffective |
| **Multimedia education** |  |  |  |  |  |  |
| Lewis 2022 | RCT;3 | Patients receive a Positive Health Check including motivational interviewing, Information-Behavior-Motivation model, and Transtheoretical Model. | Intervention (57%) vs. Control (57%), p = 1.000. | NA | NA | Effective for retention |
| Amico 2022 | RCT;12 | Remote coaching pairing for 12 weeks via secure video conferencing during clinic visits. Interact with participants via SMS and phone if the EDM pill bottle signals late or missed doses. | 12-month viral suppression: Control 35.6% vs. Intervention 34.9%, p > 0.99. | Control 72% vs. Intervention 41%, p < 0.001. | 6-month Attrition: Intervention 18.8% vs. Control 28.2%, p = 0.04. | Effective for adherence |
| Andrade-Romo 2024 | RCT;6 | Each participant receives a 4-month FUETES package, including a habit-forming toolkit with information and habit-building tools. | NA | Intervention 93.2% vs. Control 88.0%, p = 0.273. | NA | Ineffective |
| Fayorsey 2016 | RCT;24 | Assisted by aides using standardized activity charts for personalized prevention of mother-to-child transmission health education, retention, adherence support, phone and SMS appointment reminders, and follow-up for missed appointments. | Intervention 49.4% vs. SOC 40.6%, p = 0.1. | NA | NA | Effective for retention |
| Guo 2018 | RCT;9 | Weekly WeChat texts with greetings and reminders about medication adherence and regular exercise. Weekly WeChat texts include information on side effect management, medication self-management, stress management, and healthy lifestyle. | CD4+ count: Intervention 379 (254-570) vs. Control 401 (272-524), p = 0.89. | Intervention 92% vs. Control 96%, p = 0.39. | NA | Ineffective |
| **Multiple digital interventions** |  |  |  |  |  |  |
| Haberer 2016(1) | RCT;12 | Real-time adherence monitor: Scheduled SMS reminders daily for 1 month, then weekly for 2 months. For the next 6 months, SMS reminders are sent only if no monitor signal is received within 2 hours of the expected medication time. Triggered SMS: Only sent if no monitor signal is received within 2 hours of the expected medication time. | Control 90.5%, Planned SMS group 95.2%, Triggered SMS group 75%, p = 0.14. | Control 79%, Planned SMS group 91% (p = 0.02), Triggered SMS group 79% (p = 0.90). | NA | Effective for adherence |
| Kalichman 2016(2) | RCT;12 | Face-to-face office visits followed by four biweekly counseling sessions via phone. Up to two daily medication time reminders. Contact-matched non-contaminated health improvement interventions. | Adherence Counseling 79% vs. Adherence Counseling + Reminders 79% vs. Contact 79% vs. Matched Control 73% vs. Contact Matched + Reminders 76%; Adherence counseling significant, p < 0.05. SMS effect and counseling × SMS interaction not significant. | Adherence Counseling 43%, Adherence Counseling + Reminders 43%, Matched Control 42%, Contact Matched + Reminders 34%, significant adherence counseling, p < 0.05. SMS effects, counseling × SMS interaction not significant. | NA | Effective |
| Ramsey 2021 | RCT;2.5 | CBT and motivational interviewing techniques based on LifeSteps course, daily push notifications for medication reminders, and weekly check-ins. | Intervention 90.6% vs. Control 82.8%, p = 0.34. | Control (77.5, SE = 3.5) vs. Intervention (85.1, SE = 3.1), p = 0.10. | Intervention 47.27% vs. Standard treatment 37.72%. | Effective |
| Steward2021(2) | RCT;12 | Appointment reminders before the visit, and follow-up reminders every two weeks after a missed appointment until the participant returns to care or after three months. Biweekly brief behavioral information and SMS check-in messages. Navigator meets patients monthly and handles appointment scheduling via phone or SMS. | NA | NA | NA | Effective |
| Whiteley 2018 | RCT;4 | HIV-related games plus electronic medication monitoring device, receiving game-related SMS twice weekly based on monitoring device data. | Log VL: Intervention group mean (SD) 0.93 (1.63) vs. Control group 1.53 (2.49), p = 0.04. | NA | NA | Effective |
| Humans 2024 | RCT;11 | TWM is a 5-month (150-day), mobile-optimized web-based intervention that includes: 1.Peer interaction, allowing participants to post and comment on text, images, and videos in an unstructured format; 2.Brief, tailored articles on HIV and ART adherence, posted daily; 3.Daily SMS reminders, with participants able to reply by text to report same-day ART adherence and overall mood; and 4.Weekly reflections on adherence, emotional well-being, and substance use. | NA | NA | NA | Ineffective |
| Schnall 2025 | RCT;6 | An mHealth app was integrated to support self-management among people with HIV (PWH), while community health workers (CHWs) provided enhanced support and real-time monitoring of ART adherence. | Log VL: Intervention group mean (SE) 0.71 (0.14) vs. Control group 1 (0.25), p = 0.32. | NA | Intervention 70.7% vs. Controlt 77.3%. | Effective |
| Naggiriny a 2024 | [RCT;12](http://clinicaltrials.gov/show/NCT04718974) | Call for Life–IVR mHealth Group: The CFL-IVR tool is a software-based intervention built on the open-source Mobile Technology for Community Health (MoTeCH) platform. Participants received weekly health messages on sexual and reproductive health, HIV and ART, positive living, prevention of mother-to-child transmission, and HIV-related opportunistic infections. Daily medication reminders and semiannual clinic visit reminders were personalized. | NA | NA | Intervention 88.4% vs. Standard treatment 74.7%.p=0.01. | Effective |
| Mimiaga 2025 | RCT;12 | Text messaging and adherence counseling. | NA | NA | Intervention 81% vs. Controlt 86.7%. | Effective |

ART, antiretroviral therapy; IQR, Interquartile range; NA, not available; NR, not reported; RCT, randomized controlled trials; SD, standard deviation; SMS, short message service; VL, virus load.

**Table S2. Sensitivity Analysis**

| **Viral Suppression** | | | |
| --- | --- | --- | --- |
| Study omitted | Estimate | [95% Conf. | Interval] |
| Abiodun 2021 | 1.0366446 | 1.0067208 | 1.0674579 |
| Amico 2022 | 1.0406502 | 1.0077877 | 1.0745842 |
| Aunon 2021 | 1.0402391 | 1.0071577 | 1.0744071 |
| Belzer 2014 | 1.0395133 | 1.0089523 | 1.0710002 |
| Boer 2022(1) | 1.0409576 | 1.0078825 | 1.0751181 |
| Boer 2022(2) | 1.0411381 | 1.0080901 | 1.0752695 |
| Byonanebye 2021 | 1.0458243 | 1.0126426 | 1.0800933 |
| Chongpornchai 2021 | 1.041574 | 1.0087534 | 1.0754625 |
| DiPrete 2019 | 1.0416522 | 1.008504 | 1.0758898 |
| Ellsworth 2020 | 1.0396512 | 1.0071367 | 1.0732154 |
| Fayorsey 2016 | 1.0375032 | 1.0056736 | 1.0703403 |
| Garofalo 2016 | 1.0383756 | 1.0070382 | 1.070688 |
| Haberer 2016(1) | 1.0422217 | 1.0107783 | 1.0746433 |
| Haberer 2016(2) | 1.0401661 | 1.0069845 | 1.0744412 |
| Kalichman 2016 | 1.0436398 | 1.0109372 | 1.0774001 |
| Kalichman 2022 | 1.0405297 | 1.0072665 | 1.0748914 |
| Kiruthu-Kamamia 2025 | 1.0404432 | 1.0036271 | 1.0786098 |
| Lester 2010 | 1.035769 | 1.0042765 | 1.0682491 |
| Lewis 2022 | 1.0399345 | 1.0065624 | 1.0744129 |
| Liu 2022 | 1.0370342 | 1.005556 | 1.0694977 |
| Orrell 2015 | 1.0402433 | 1.0062535 | 1.0753813 |
| Ramsey 2021 | 1.039396 | 1.0063893 | 1.0734854 |
| Ruel2023 | 1.0267872 | 0.99686015 | 1.0576127 |
| Saberi2021 | 1.0388845 | 1.0061731 | 1.0726594 |
| Sabin2015 | 1.0499927 | 1.0205581 | 1.0802763 |
| Schnall 2025 | 1.043017 | 1.0099715 | 1.0771438 |
| Sherman2020 | 1.0395578 | 1.0064744 | 1.0737286 |
| Shet2014 | 1.0452651 | 1.0108941 | 1.0808047 |
| Uzma 2011 | 1.0405999 | 1.0073304 | 1.0749685 |
| Combined | 1.0402354 | 1.008143 | 1.0733495 |
| **CD4⁺ Cell Counts** | | | |
| Abdulrahman 2017 | 0.06816446 | -0.05089112 | 0.18722004 |
| Claborn 2014 | 0.16785443 | 0.0558638 | 0.27984506 |
| Guo 2018 | 0.14461179 | 0.03452775 | 0.25469583 |
| Huang 2013(1) | 0.13491404 | 0.0237232 | 0.24610488 |
| Huang 2013(2) | 0.14792456 | 0.03746362 | 0.25838551 |
| Mbuagbaw 2012 | 0.1309476 | 0.01388103 | 0.24801418 |
| Ruan 2017 | 0.15085796 | 0.03867044 | 0.26304549 |
| Sabin2015 | 0.1128426 | -0.00014095 | 0.22582616 |
| Sherman 2020 | 0.13975073 | 0.02784001 | 0.25166145 |
| Simoni 2009 | 0.16352563 | 0.04516407 | 0.28188717 |
| Trinidad 2025 | 0.08384214 | -0.02665907 | 0.19434334 |
| Combined | 0.13174715 | 0.02388455 | 0.23960974 |
| **Subjective adherence** | | | |
| Abdulrahman 2017 | 1.0898267 | 1.0250957 | 1.1586452 |
| Abiodun 2021 | 1.1259001 | 1.0452145 | 1.2128145 |
| Abuogi 2022 | 1.1417027 | 1.0522722 | 1.2387336 |
| Andrade-Romo 2024 | 1.1386675 | 1.0551089 | 1.2288435 |
| Aunon 2021 | 1.131474 | 1.0487837 | 1.2206841 |
| Ayer 2021 | 1.1331512 | 1.0470324 | 1.2263533 |
| Belzer 2014 | 1.1228147 | 1.0458505 | 1.2054425 |
| Boer 2022 | 1.1402397 | 1.0554622 | 1.2318268 |
| Claborn 2014 | 1.1328714 | 1.0526025 | 1.2192614 |
| Costa 2012(1) | 1.1279544 | 1.0470598 | 1.2150987 |
| Ellsworth 2020 | 1.1287632 | 1.0492429 | 1.2143102 |
| Garofalo 2016 | 1.1270937 | 1.0467739 | 1.2135764 |
| Guo 2018 | 1.1411488 | 1.0578871 | 1.2309637 |
| Jiao 2022 | 1.1274478 | 1.043184 | 1.2185181 |
| Kim 2019(1) | 1.1476951 | 1.0726534 | 1.2279865 |
| Lester 2010 | 1.1220968 | 1.0411159 | 1.2093766 |
| Lizárraga 2019 | 1.1114364 | 1.0351835 | 1.1933061 |
| Mbuagbaw 2012 | 1.1325362 | 1.0499389 | 1.2216313 |
| Moore 2012 | 1.1252216 | 1.0458398 | 1.2106286 |
| Ruan 2017 | 1.1232444 | 1.0424716 | 1.2102755 |
| Saberi 2021 | 1.1258777 | 1.0468512 | 1.21087 |
| Simoni 2009 | 1.1241467 | 1.0437658 | 1.2107178 |
| Uzma 2011 | 1.1298882 | 1.0479307 | 1.2182554 |
| Combined | 1.128193 | 1.0493753 | 1.2129306 |
| **Objective adherence** | | | |
| Study omitted | Estimate | [95% Conf. | Interval] |
| Costa 2012(2) | 1.1847481 | 0.99966532 | 1.4040978 |
| DeFulio 2021 | 1.1497068 | 0.98059404 | 1.3479849 |
| Kalichman 2016 | 1.2476579 | 1.0404066 | 1.4961941 |
| Linnemayr 2017 | 1.2172415 | 1.0174116 | 1.4563199 |
| Maduka 2013 | 1.1629205 | 0.976174 | 1.3853923 |
| Pop-Eleches 2011 | 1.1933964 | 0.99080604 | 1.4374106 |
| Sherman 2020 | 1.2017294 | 0.99366701 | 1.4533577 |
| Shet 2014 | 1.2396318 | 1.03671 | 1.4822727 |
| Trinidad 2025 | 1.121702 | 0.96670026 | 1.3015568 |
| Kiruthu-Kamamia 2025 | 1.1666472 | 0.97562414 | 1.3950716 |
| Combined | 1.1857408 | 1.0049122 | 1.3991085 |
| **Retention** | | | |
| Abuogi 2022 | 1.0645856 | 1.0095652 | 1.1226046 |
| Byonanebye 2021 | 1.0644652 | 1.0107158 | 1.1210728 |
| Christopoulos 2018 | 1.0673006 | 1.0140668 | 1.1233288 |
| Davey 2016 | 1.063825 | 1.0070066 | 1.1238492 |
| Dulli 2020 | 1.0693617 | 1.0162667 | 1.1252307 |
| Elul 2017 | 1.0488285 | 1.0028793 | 1.0968829 |
| Fayorsey 2016 | 1.0606941 | 1.0071393 | 1.1170968 |
| Hasin 2021 | 1.0635693 | 1.0101616 | 1.1198008 |
| Huang 2013 | 1.0634339 | 1.0093074 | 1.1204631 |
| Humans 2024 | 1.0690366 | 1.0159256 | 1.1249241 |
| Ingersoll 2015 | 1.0594765 | 1.0066707 | 1.1150522 |
| Kalichman 2022 | 1.0626243 | 1.0090096 | 1.1190881 |
| Ketchaji 2025 | 1.0643346 | 1.0094067 | 1.1222516 |
| Kim 2019 | 1.0596373 | 1.0062404 | 1.1158677 |
| Kiruthu-Kamamia 2025 | 1.0652853 | 1.0105557 | 1.122979 |
| Lester 2010 | 1.0634027 | 1.0092489 | 1.1204622 |
| Lewis 2022 | 1.0631652 | 1.0092968 | 1.1199088 |
| Mbuagbaw 2012 | 1.0668446 | 1.0133781 | 1.123132 |
| McNairy 2017 | 1.0510639 | 1.0028924 | 1.1015493 |
| Mimiaga 2025 | 1.0668998 | 1.0135392 | 1.1230696 |
| Naggirinya 2024 | 1.059341 | 1.006085 | 1.1154158 |
| Orrell 2015 | 1.0694323 | 1.0163516 | 1.1252851 |
| Pop-Eleches 2011 | 1.0619406 | 1.0092983 | 1.1173286 |
| Ruel 2023 | 1.0559309 | 1.0047272 | 1.109744 |
| Sabin2020 | 1.0650156 | 1.0120995 | 1.1206983 |
| Sarna 2019 | 1.0571915 | 1.0043229 | 1.1128432 |
| Satyanarayana 2024 | 1.0663924 | 1.0127366 | 1.1228909 |
| Schnall 2025 | 1.0679396 | 1.0146152 | 1.1240667 |
| Sherman 2020 | 1.0583228 | 1.0053331 | 1.1141055 |
| Steward 2021 | 1.0587677 | 1.0059652 | 1.1143416 |
| Tarantino 2024 | 1.0665265 | 1.0133048 | 1.1225437 |
| Trinidad 2025 | 1.0648293 | 1.0113131 | 1.1211776 |
| van der kop 2018 | 1.0661342 | 1.0120639 | 1.1230932 |
| Combined | 1.062907 | 1.0105413 | 1.1179863 |
